# Supplementary figures and images for: A comprehensive approach to characterize navigation instruments for magnetic guidance in biological systems
Source: Sci Rep. 2024 Apr 3;14:7879. doi: 10.1038/s41598-024-58091-x (PMC10991419; doi:10.1038/s41598-024-58091-x)

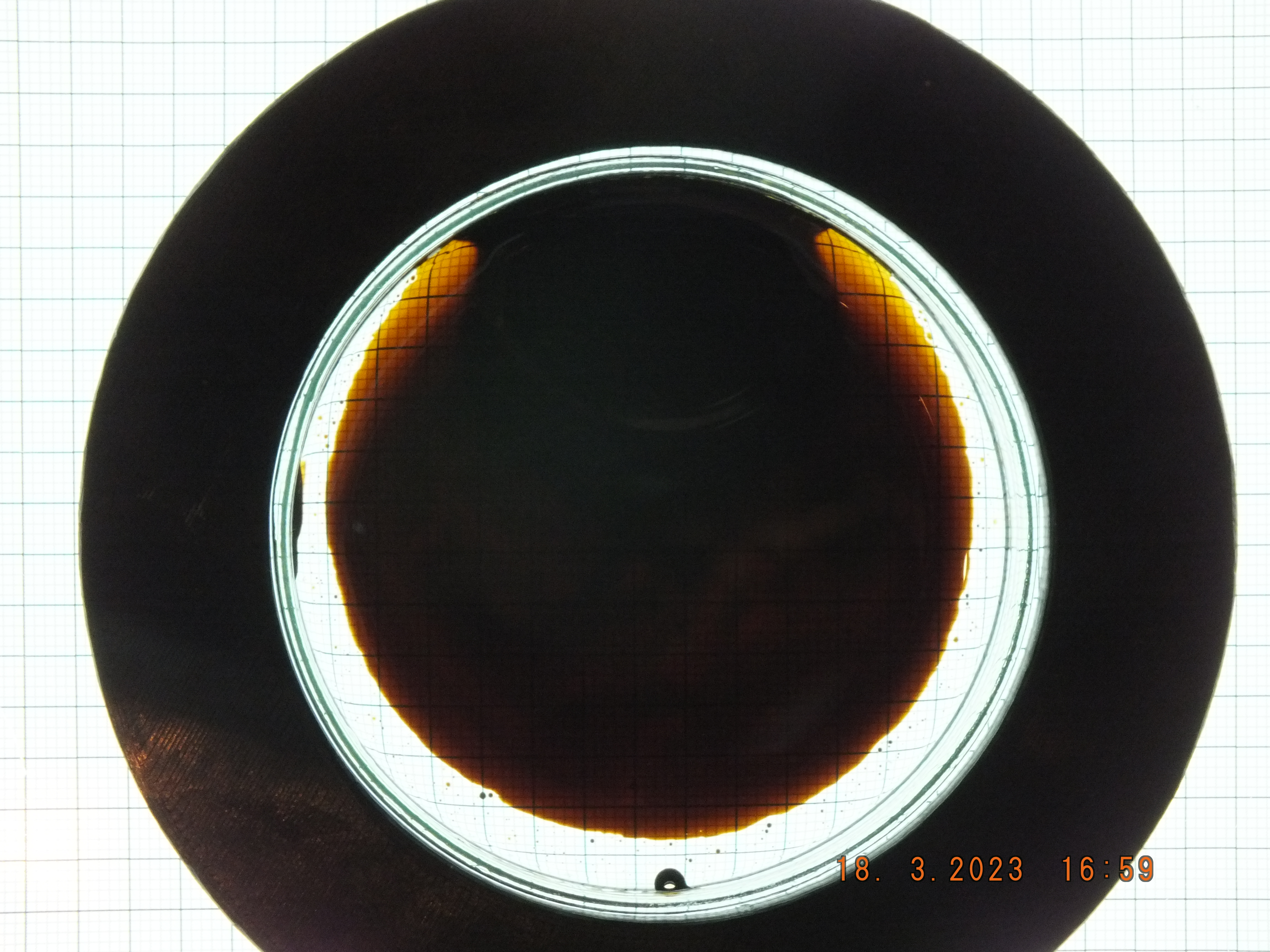

Supplement: Supplementary file 1 — Supplementary Information. [file 41598_2024_58091_MOESM1_ESM.zip › rawdata/fig6/ausgang.JPG]

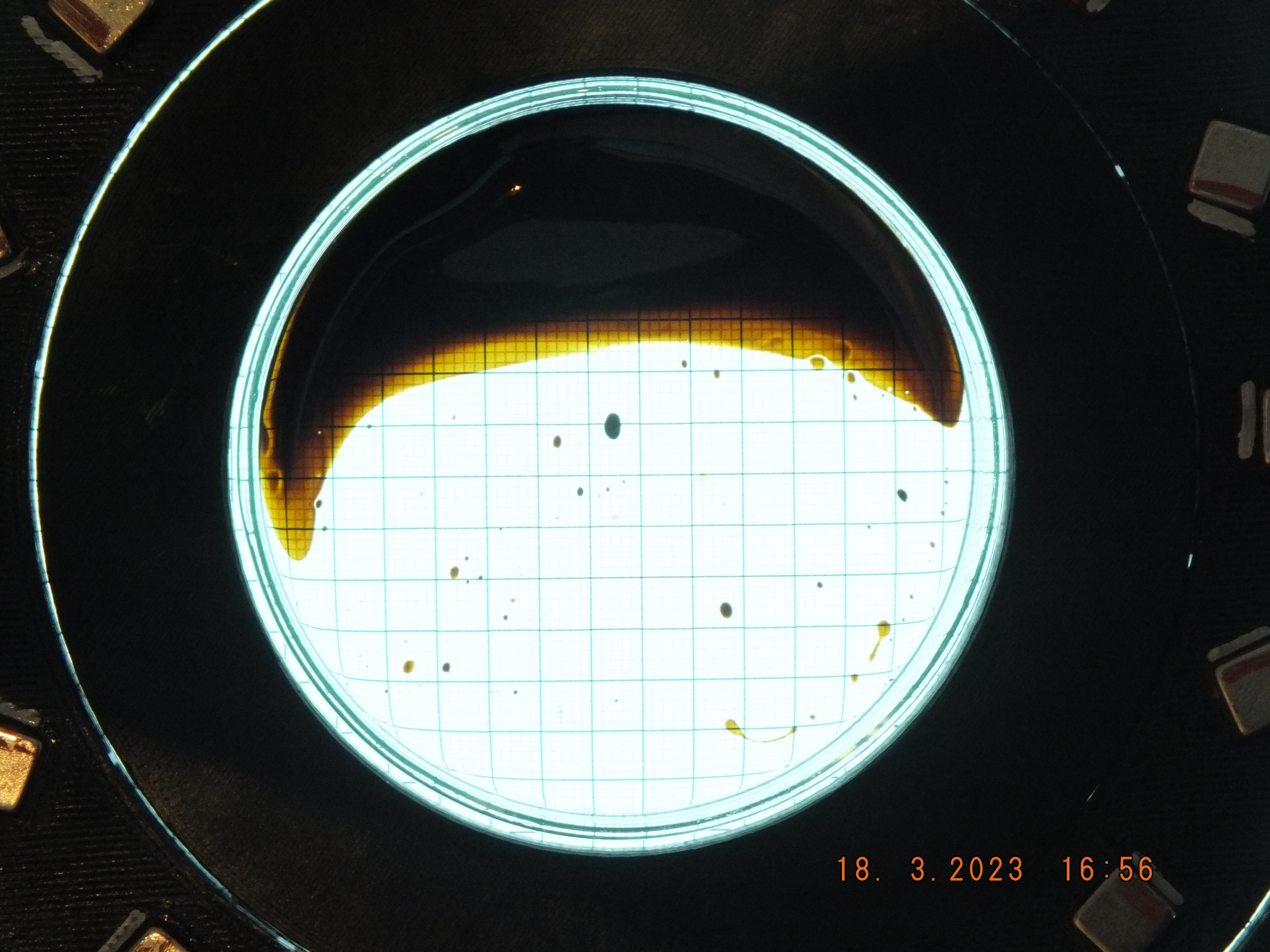

Supplement: Supplementary file 1 — Supplementary Information. [file 41598_2024_58091_MOESM1_ESM.zip › rawdata/fig6/dipol.JPG]

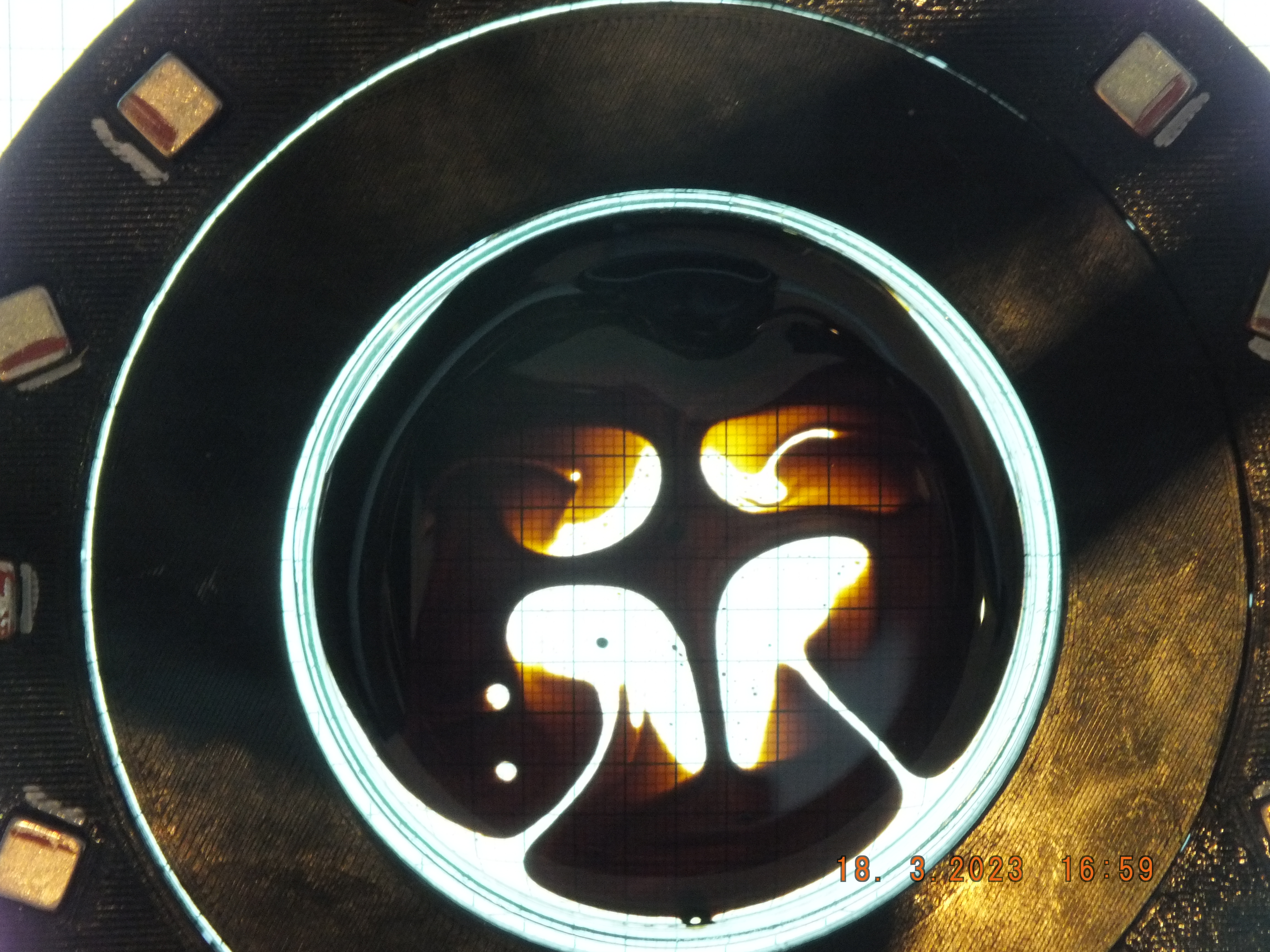

Supplement: Supplementary file 1 — Supplementary Information. [file 41598_2024_58091_MOESM1_ESM.zip › rawdata/fig6/quadrupol1.JPG]

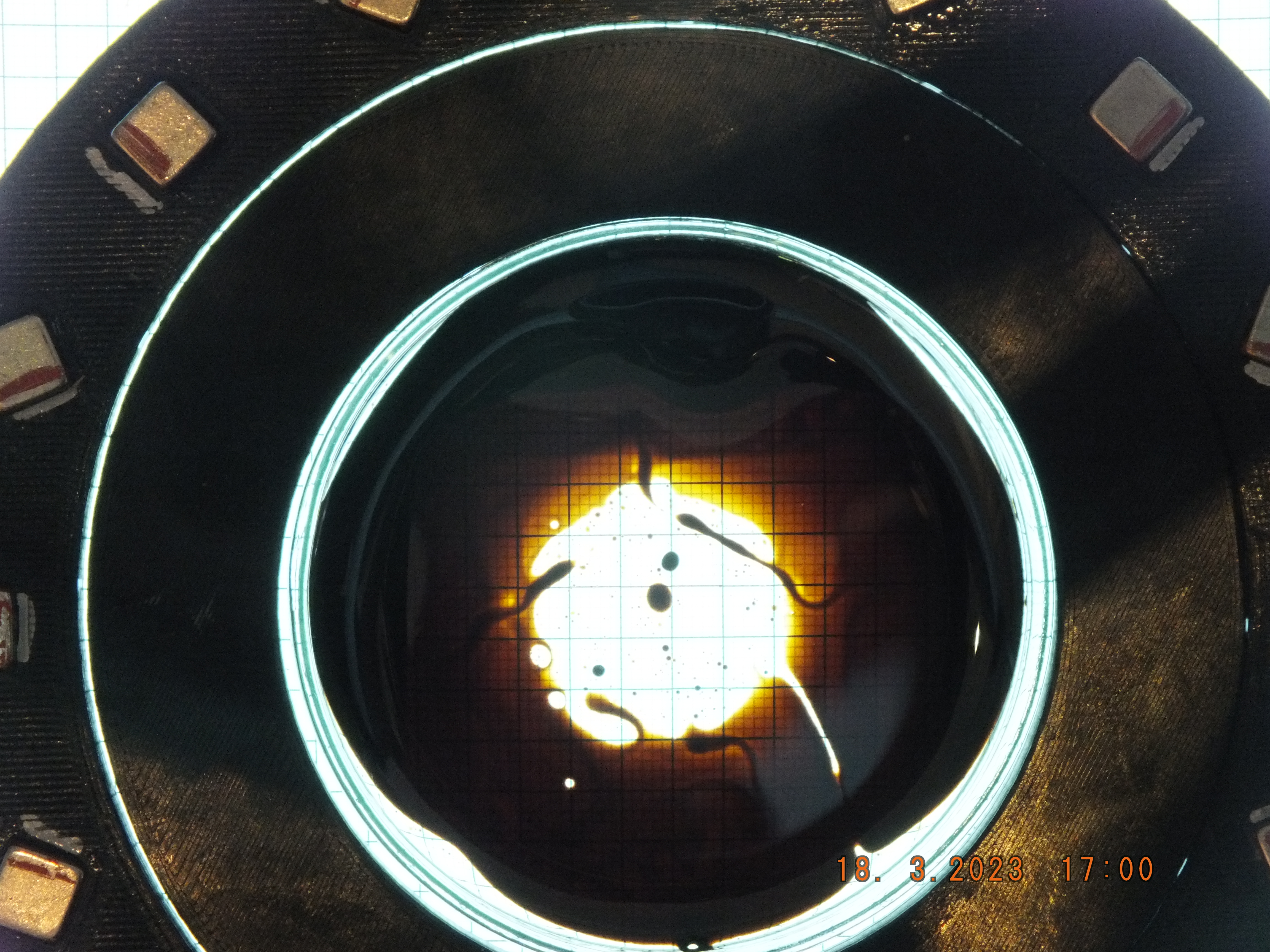

Supplement: Supplementary file 1 — Supplementary Information. [file 41598_2024_58091_MOESM1_ESM.zip › rawdata/fig6/quadrupol2.JPG]

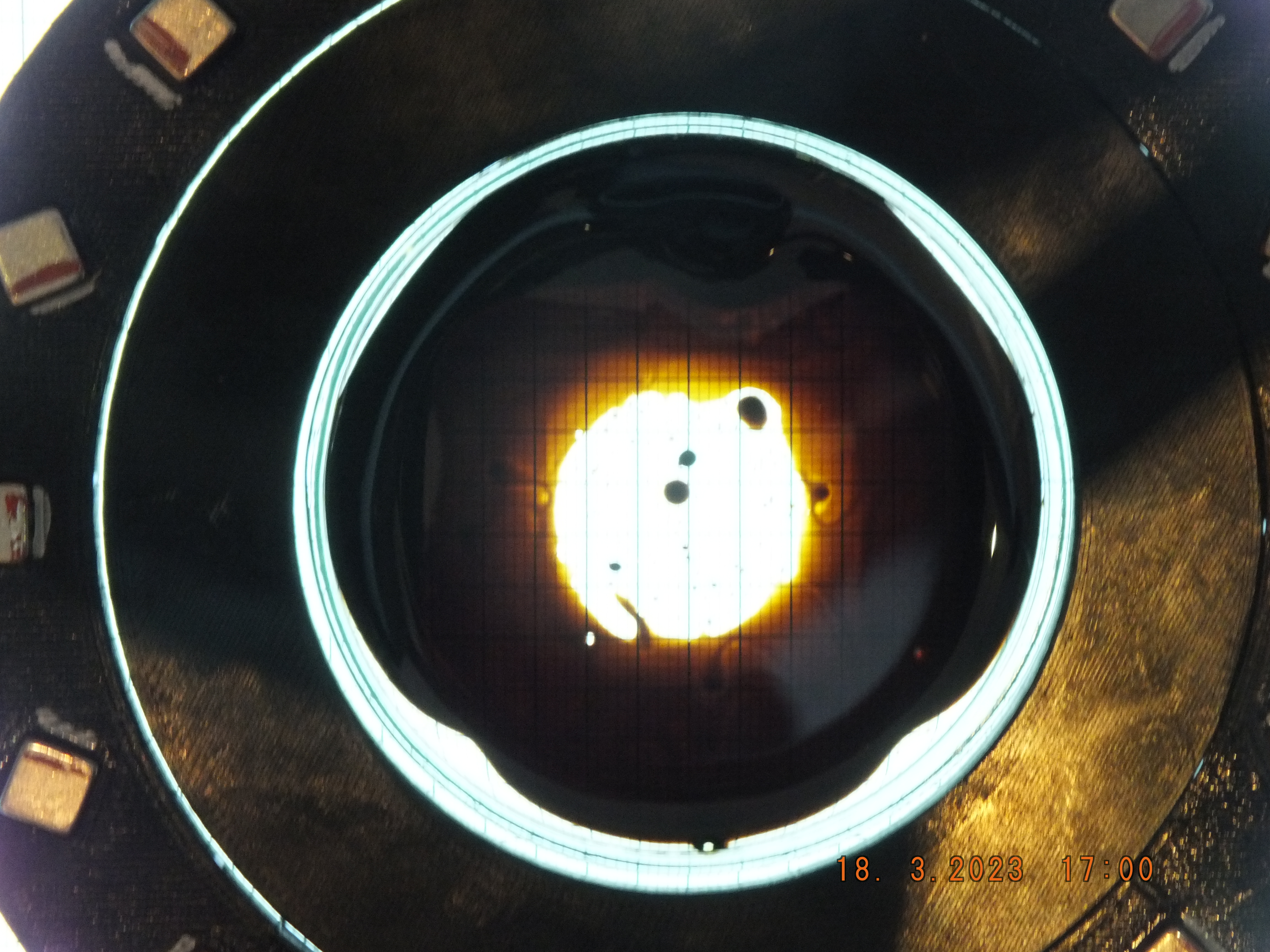

Supplement: Supplementary file 1 — Supplementary Information. [file 41598_2024_58091_MOESM1_ESM.zip › rawdata/fig6/quadrupol3.JPG]

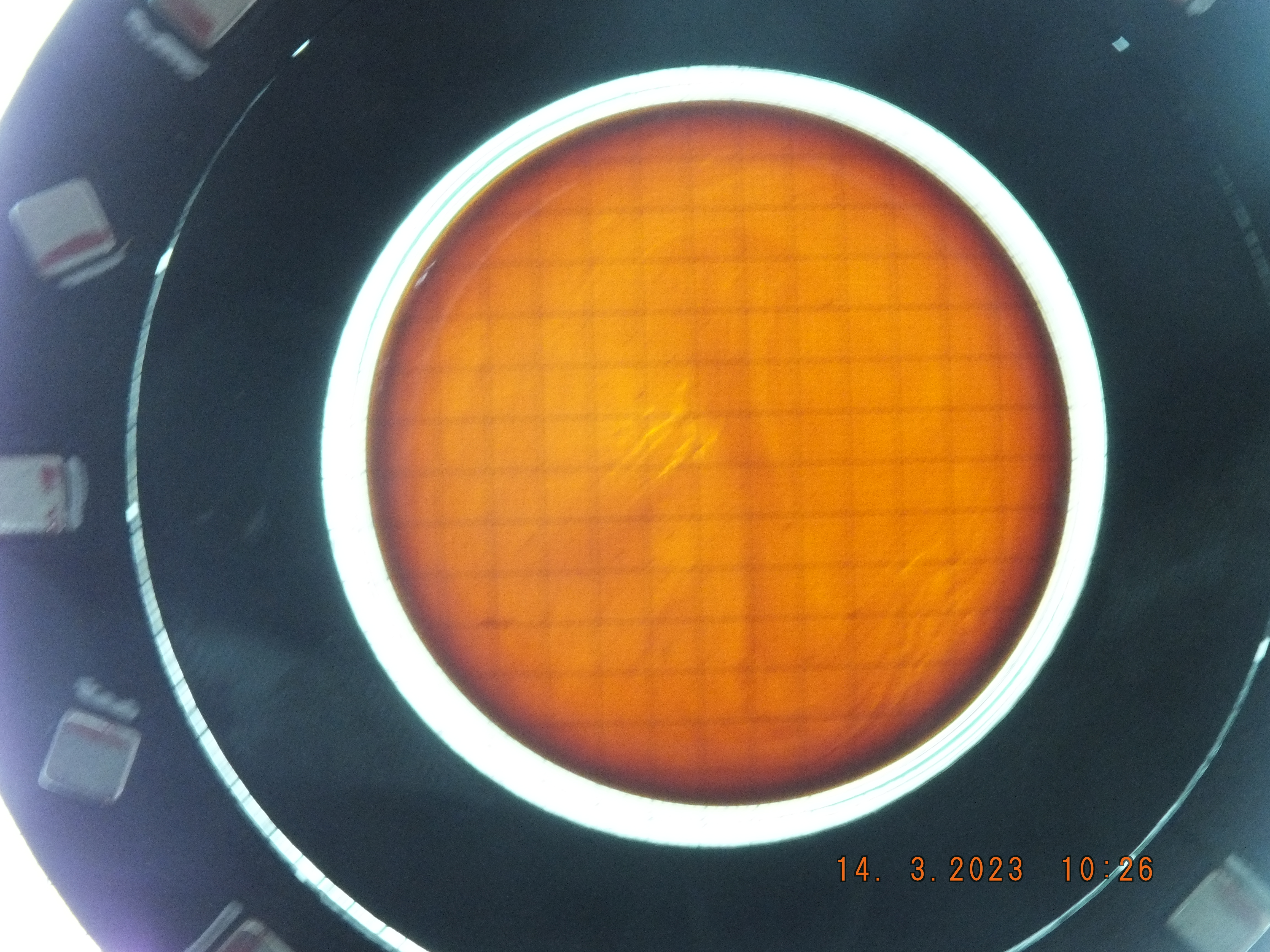

Supplement: Supplementary file 1 — Supplementary Information. [file 41598_2024_58091_MOESM1_ESM.zip › rawdata/fig7a/0.JPG]

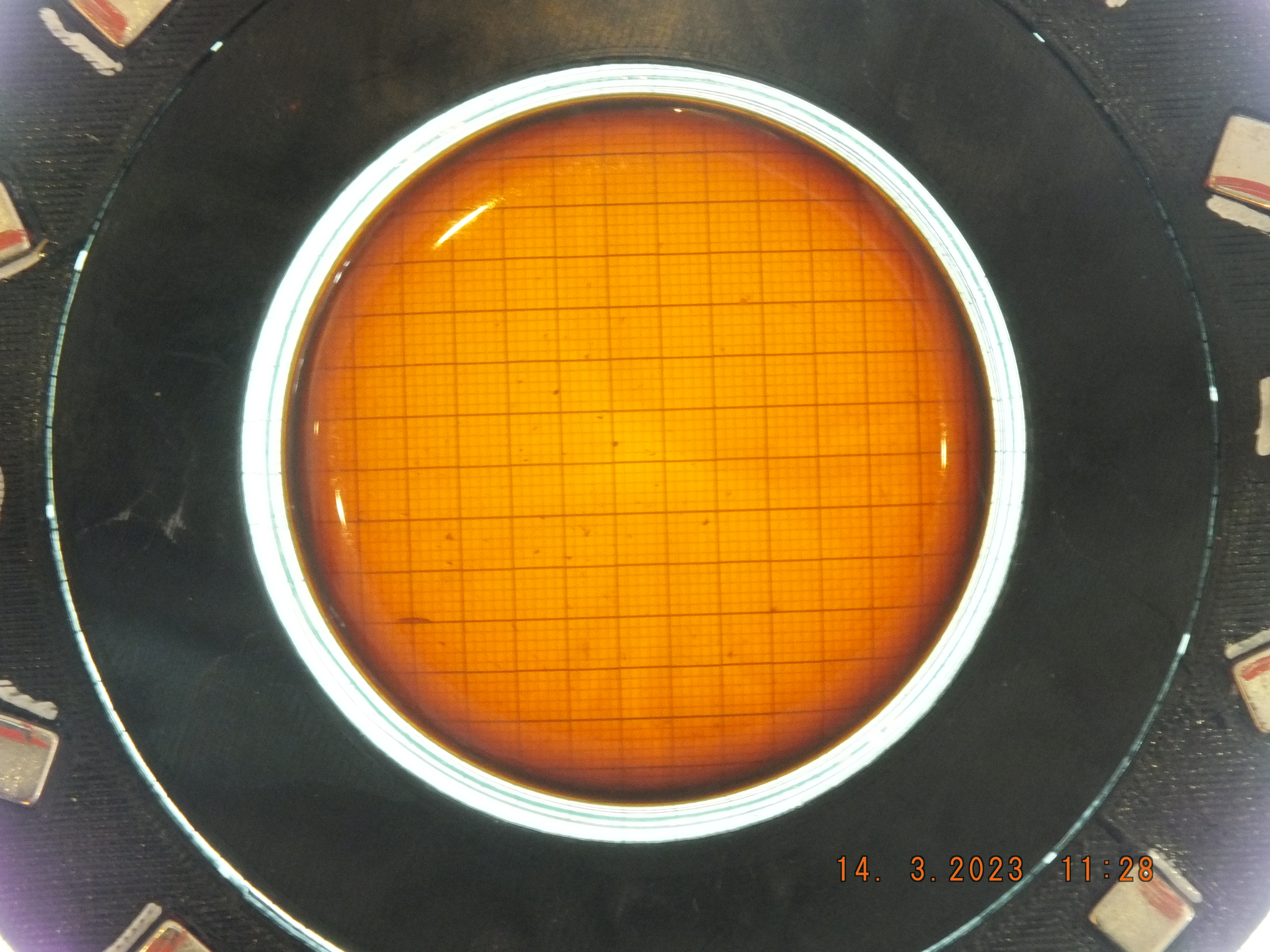

Supplement: Supplementary file 1 — Supplementary Information. [file 41598_2024_58091_MOESM1_ESM.zip › rawdata/fig7a/0_02.JPG]

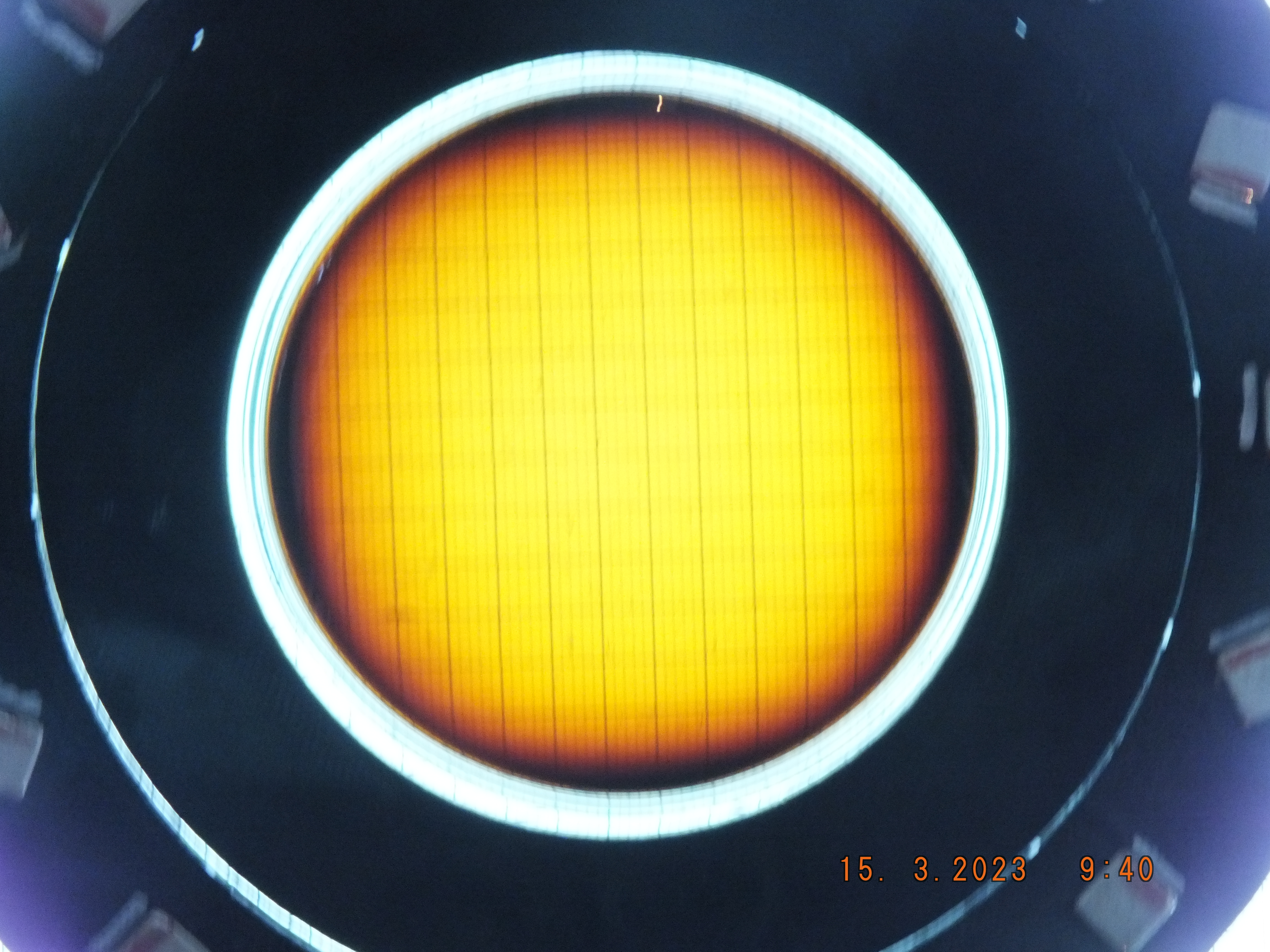

Supplement: Supplementary file 1 — Supplementary Information. [file 41598_2024_58091_MOESM1_ESM.zip › rawdata/fig7a/23_14.JPG]

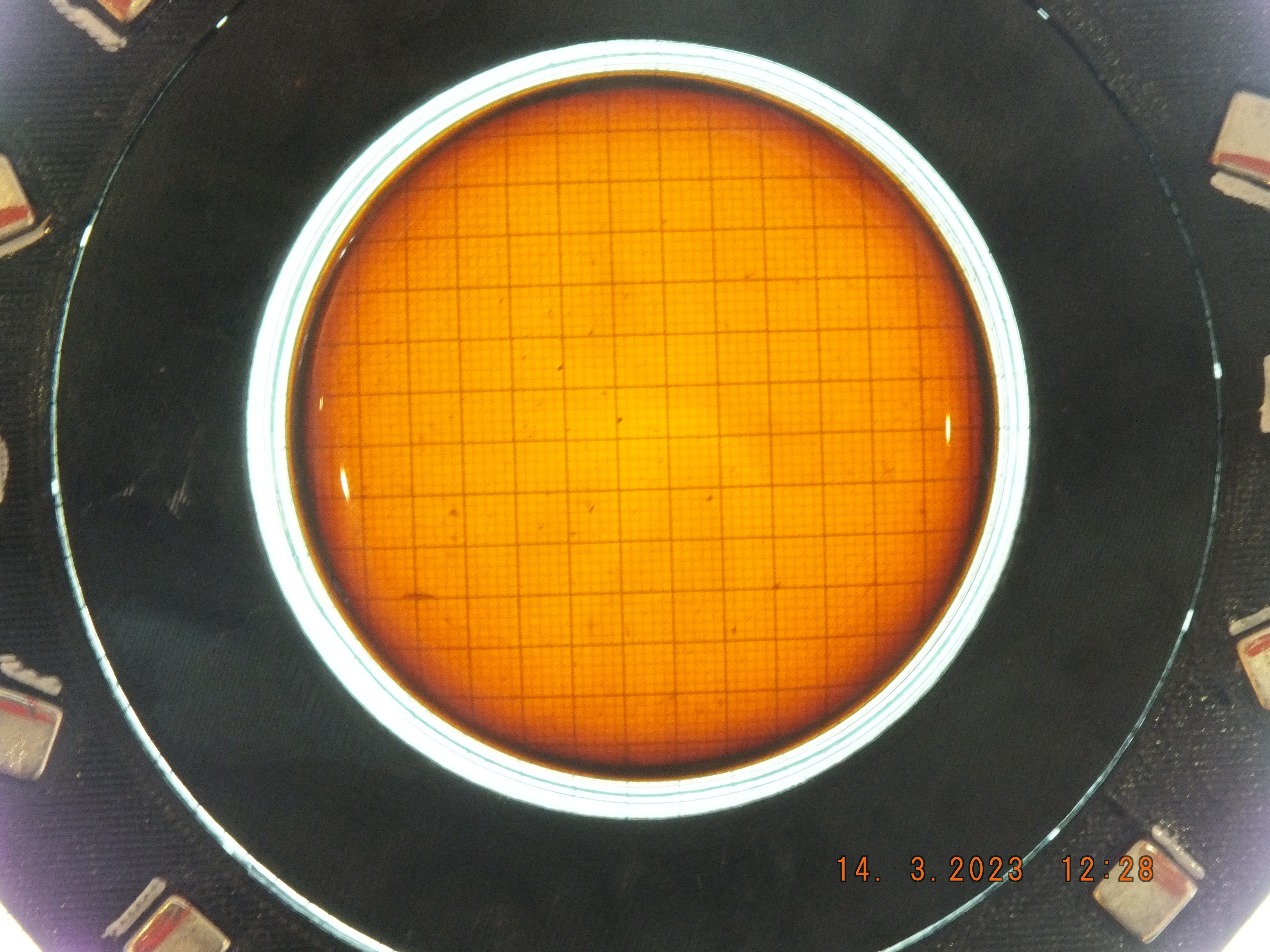

Supplement: Supplementary file 1 — Supplementary Information. [file 41598_2024_58091_MOESM1_ESM.zip › rawdata/fig7a/2_02.JPG]

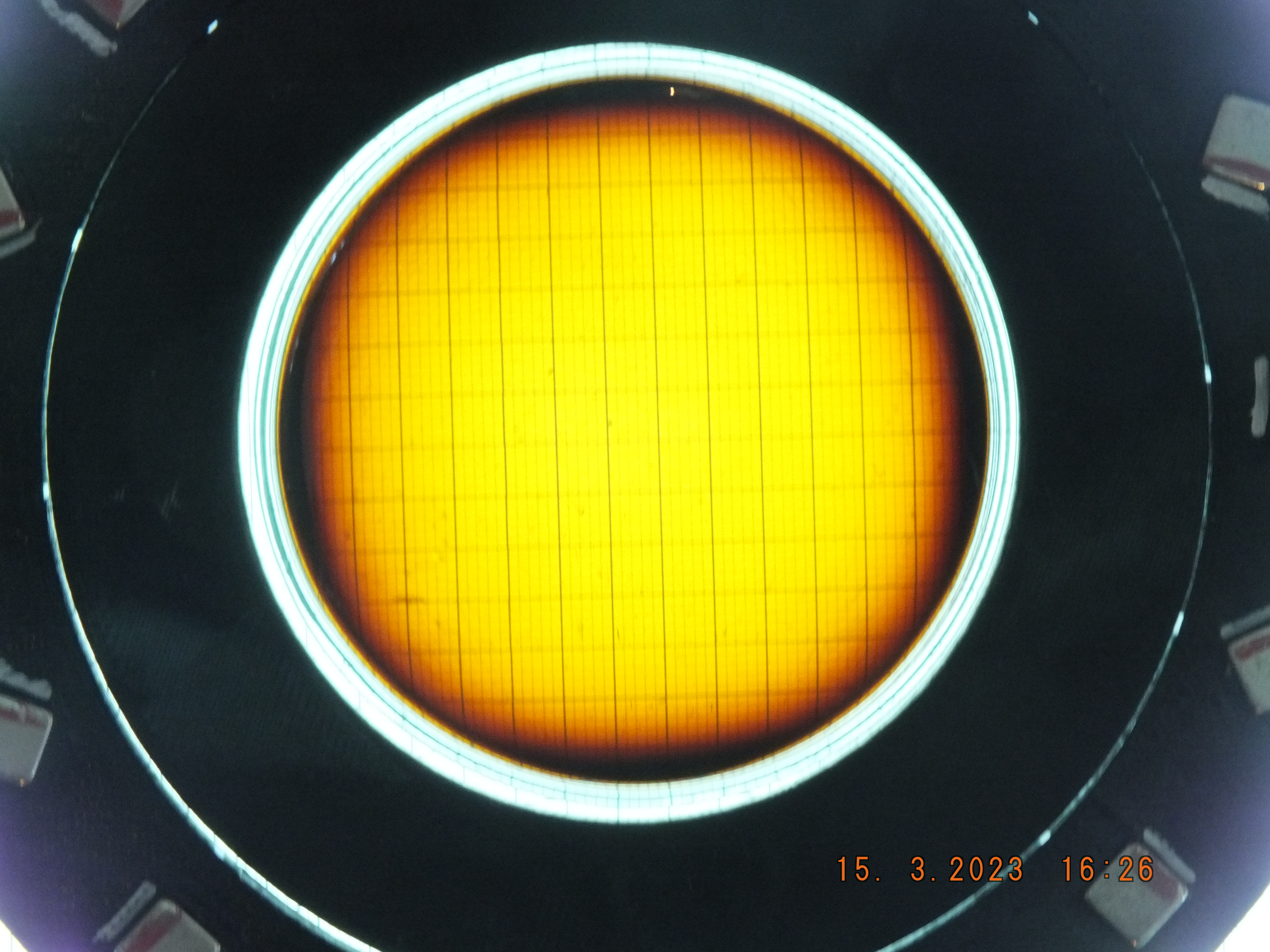

Supplement: Supplementary file 1 — Supplementary Information. [file 41598_2024_58091_MOESM1_ESM.zip › rawdata/fig7a/30_0.JPG]

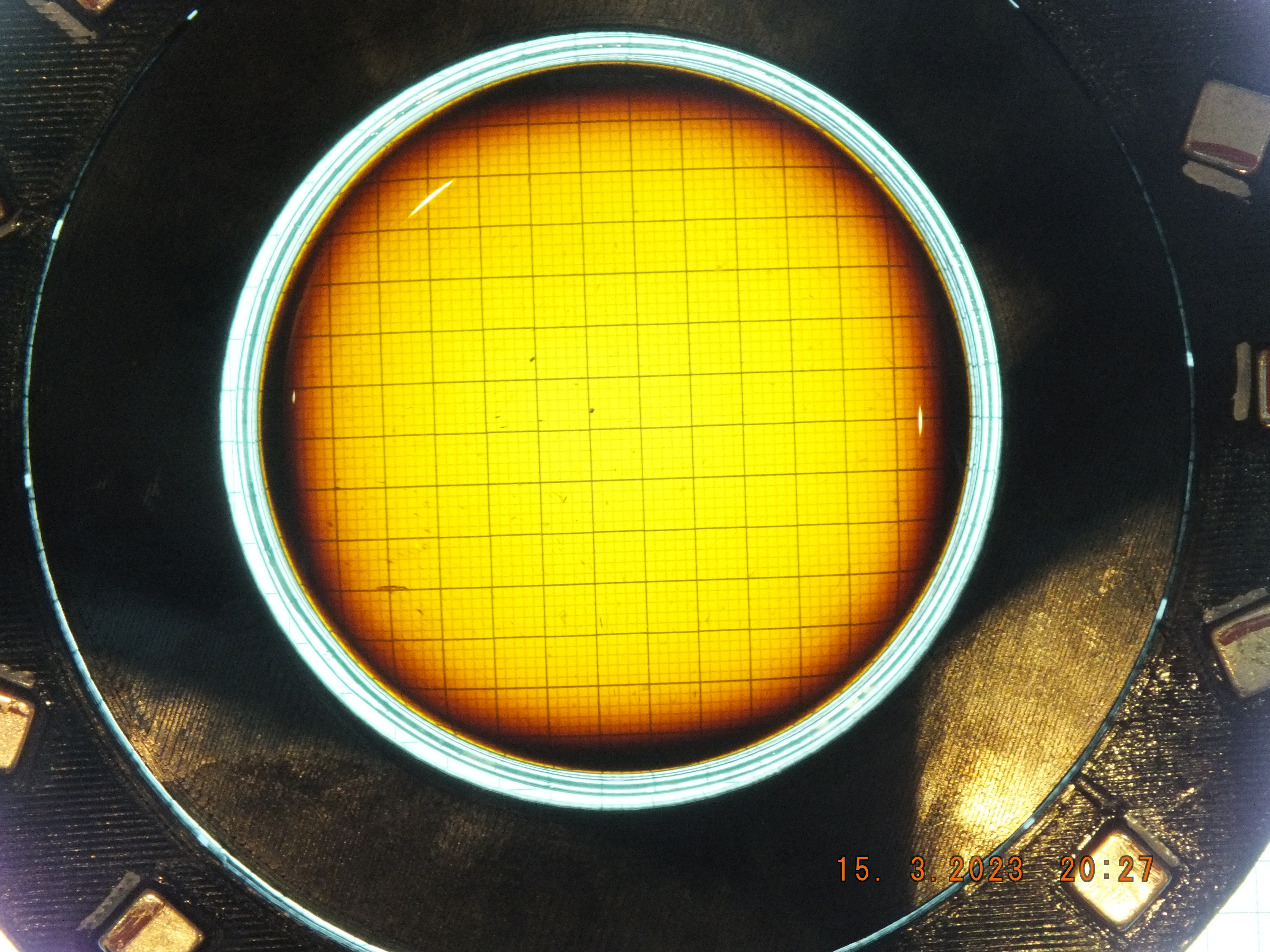

Supplement: Supplementary file 1 — Supplementary Information. [file 41598_2024_58091_MOESM1_ESM.zip › rawdata/fig7a/34_01.JPG]

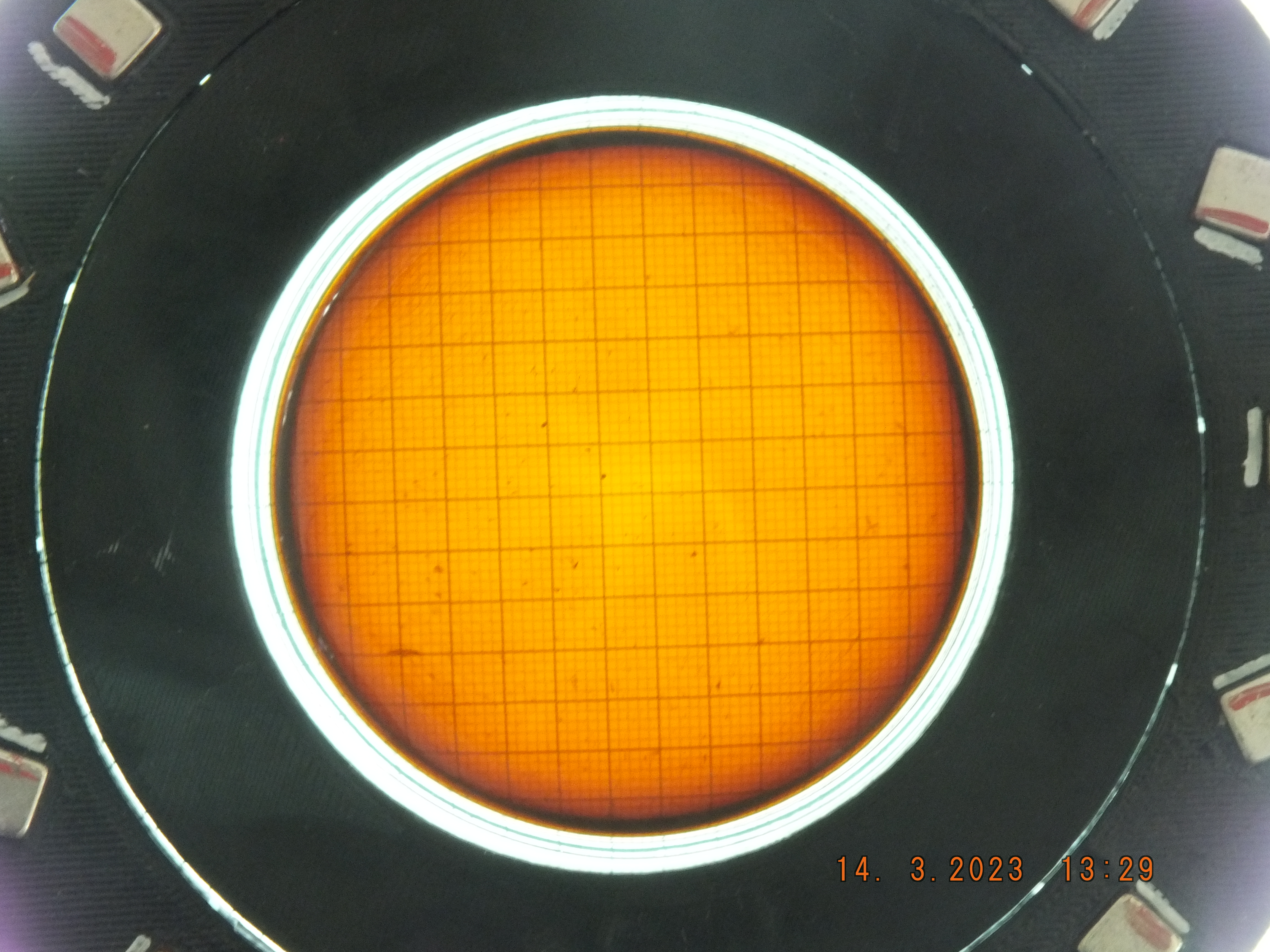

Supplement: Supplementary file 1 — Supplementary Information. [file 41598_2024_58091_MOESM1_ESM.zip › rawdata/fig7a/3_03.JPG]

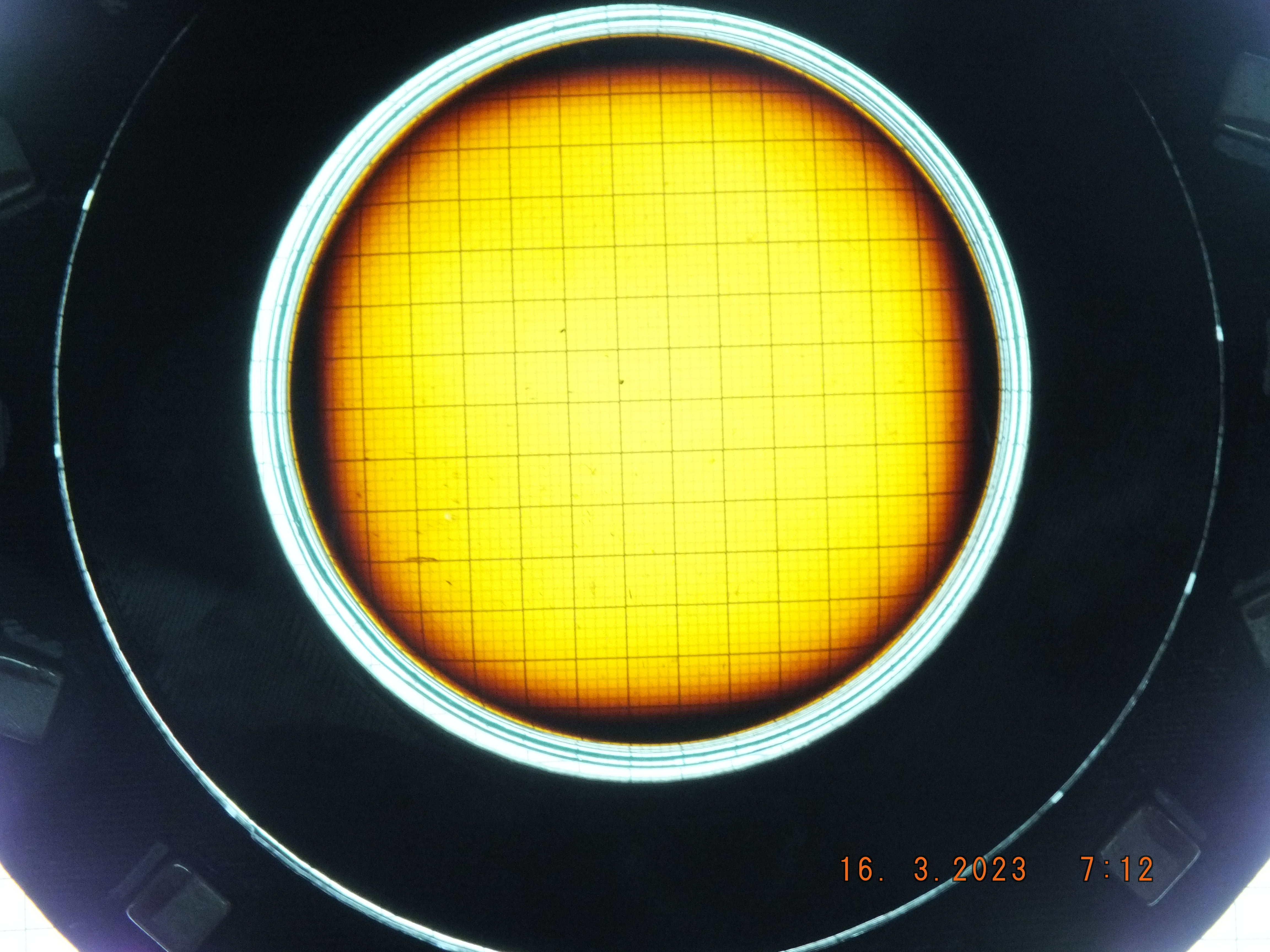

Supplement: Supplementary file 1 — Supplementary Information. [file 41598_2024_58091_MOESM1_ESM.zip › rawdata/fig7a/44_46.JPG]

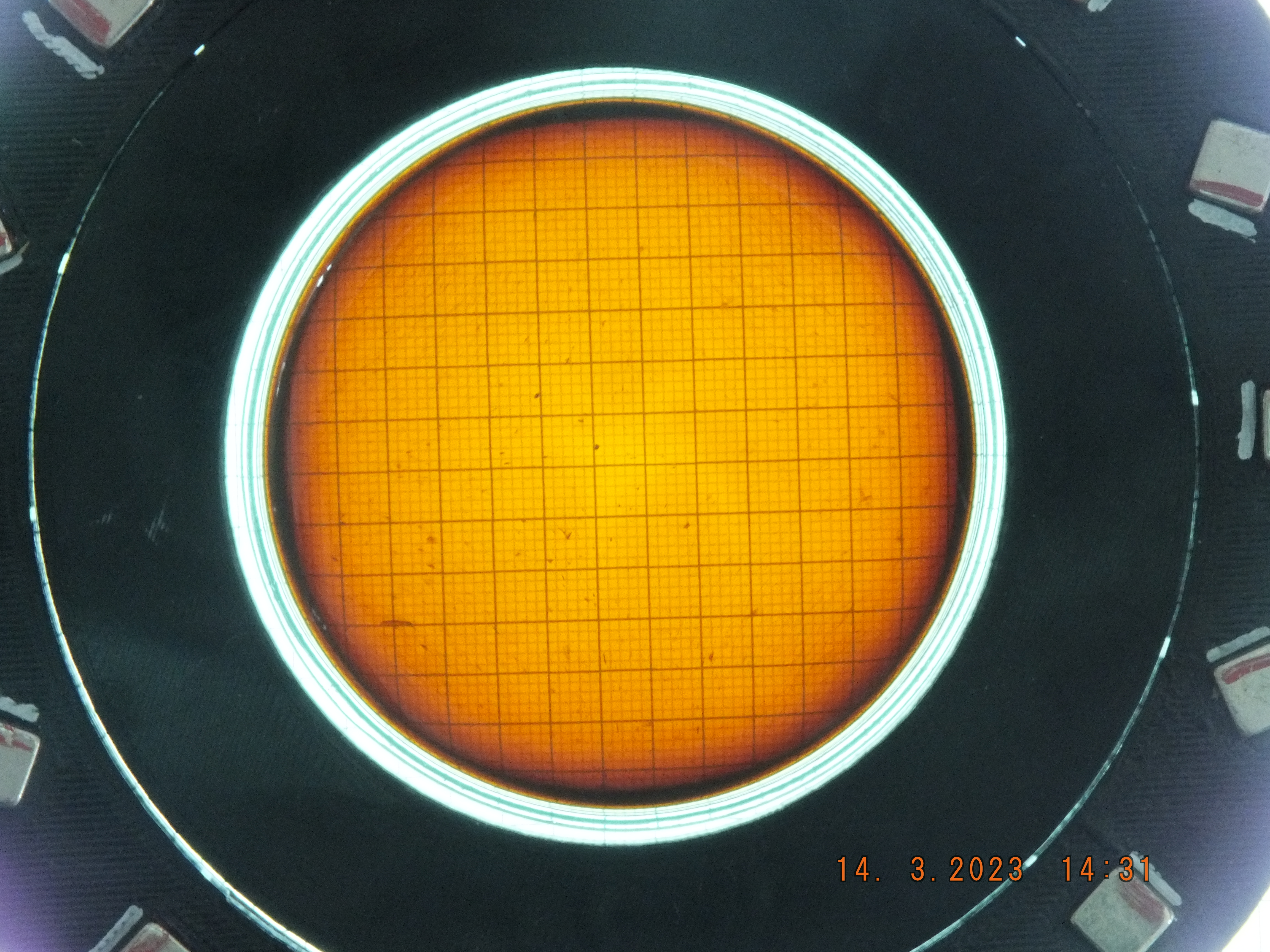

Supplement: Supplementary file 1 — Supplementary Information. [file 41598_2024_58091_MOESM1_ESM.zip › rawdata/fig7a/4_05.JPG]

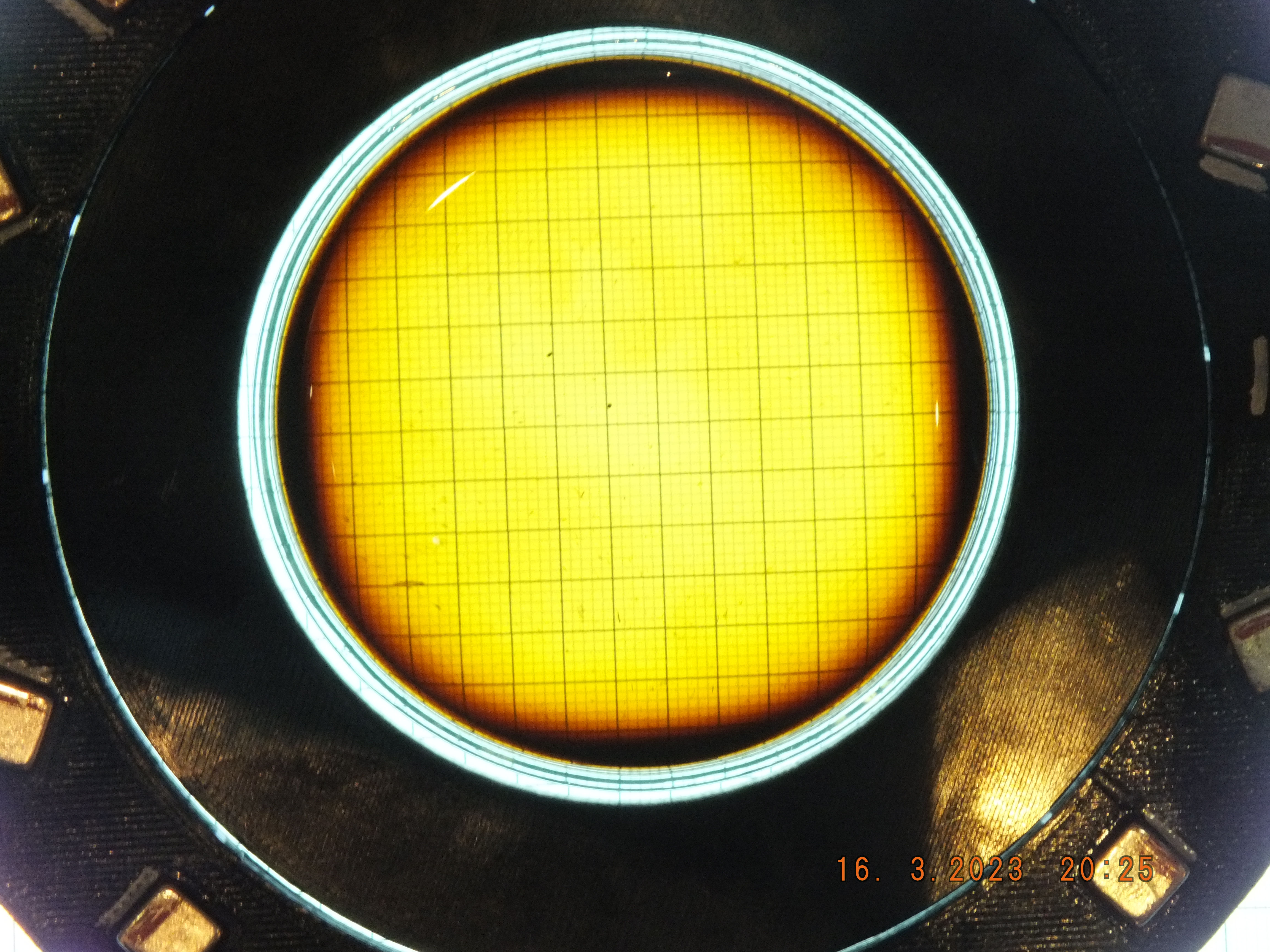

Supplement: Supplementary file 1 — Supplementary Information. [file 41598_2024_58091_MOESM1_ESM.zip › rawdata/fig7a/57_59.JPG]

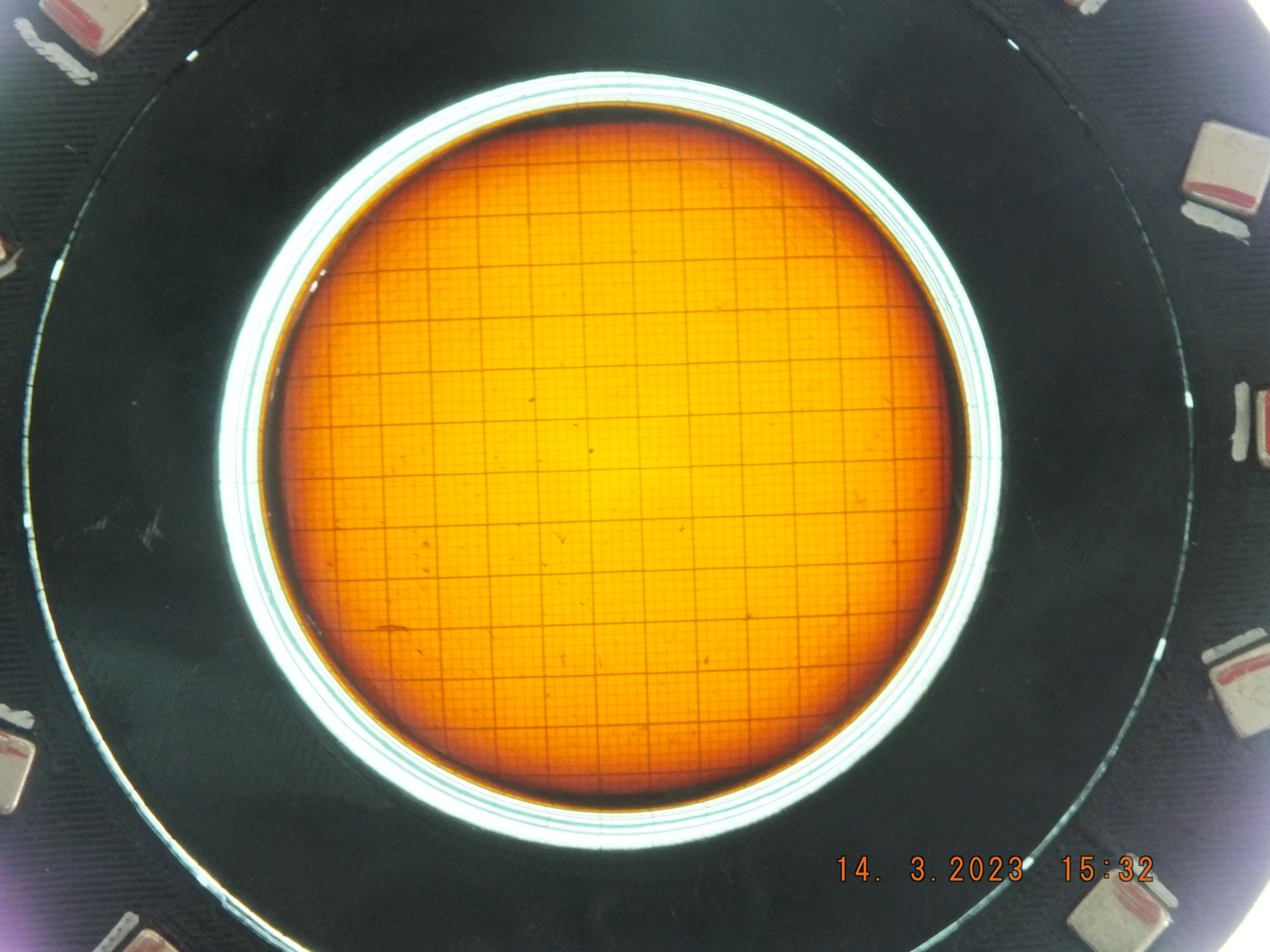

Supplement: Supplementary file 1 — Supplementary Information. [file 41598_2024_58091_MOESM1_ESM.zip › rawdata/fig7a/5_06.JPG]

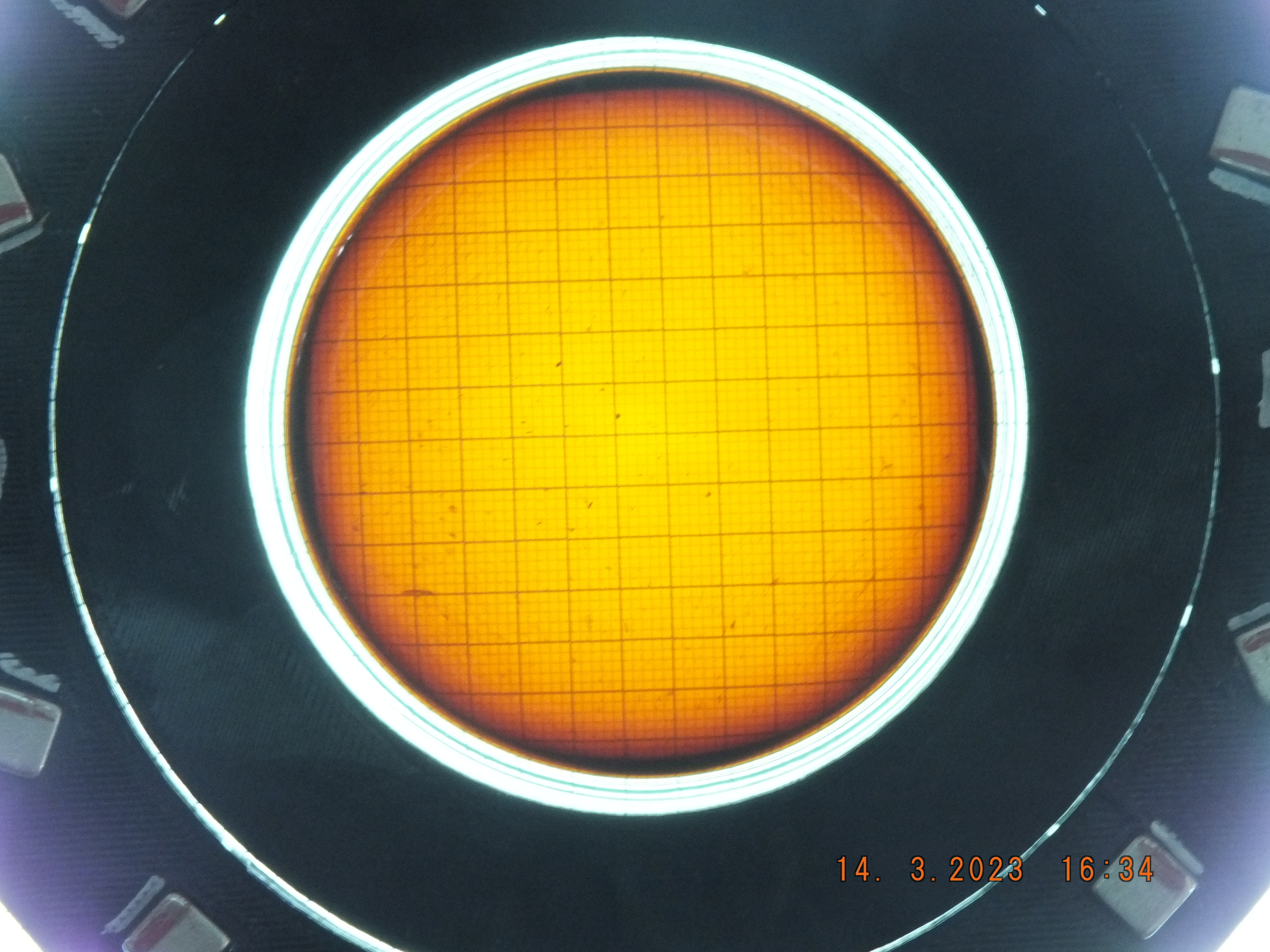

Supplement: Supplementary file 1 — Supplementary Information. [file 41598_2024_58091_MOESM1_ESM.zip › rawdata/fig7a/6_08.JPG]

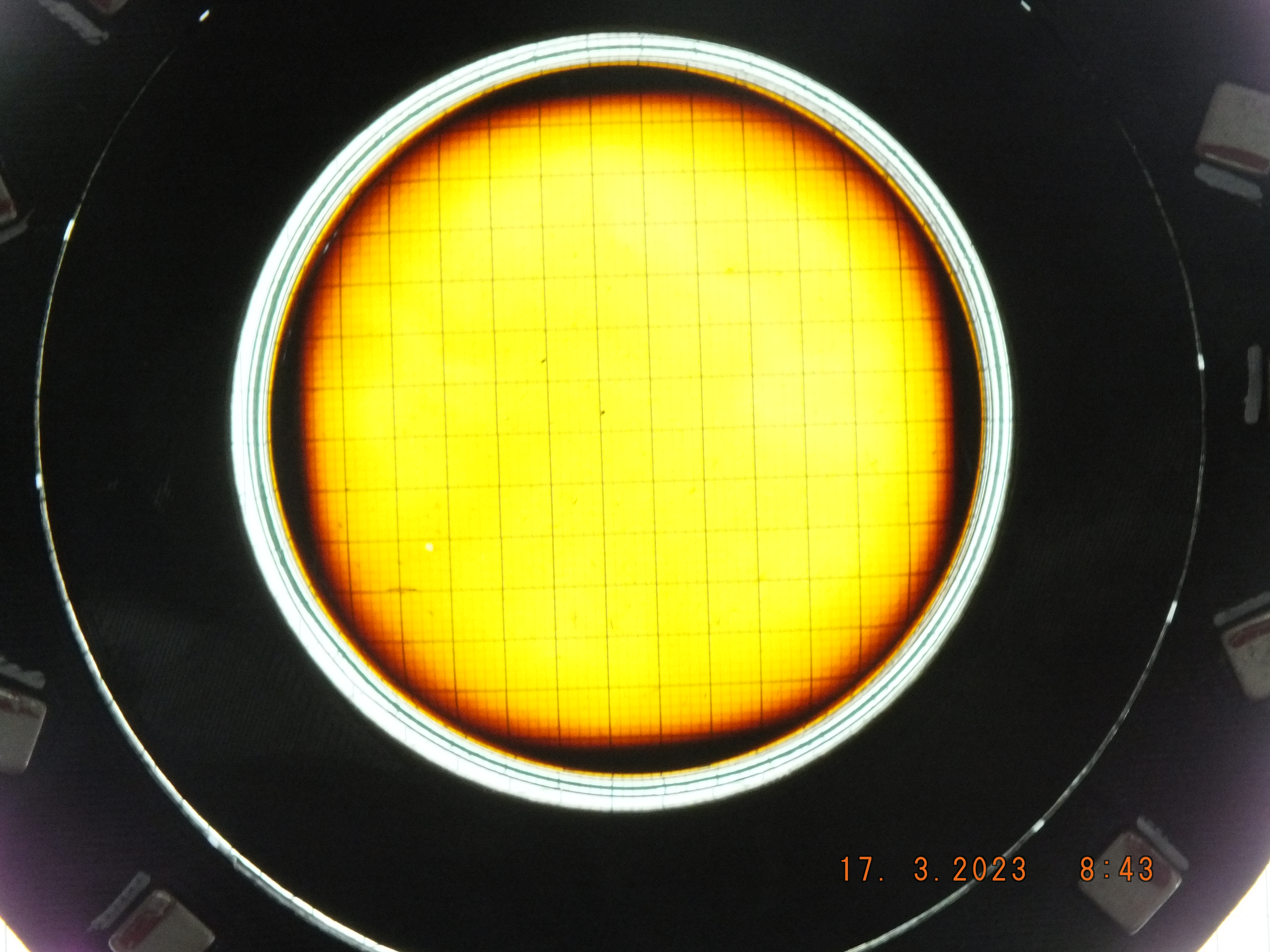

Supplement: Supplementary file 1 — Supplementary Information. [file 41598_2024_58091_MOESM1_ESM.zip › rawdata/fig7a/70_17.JPG]

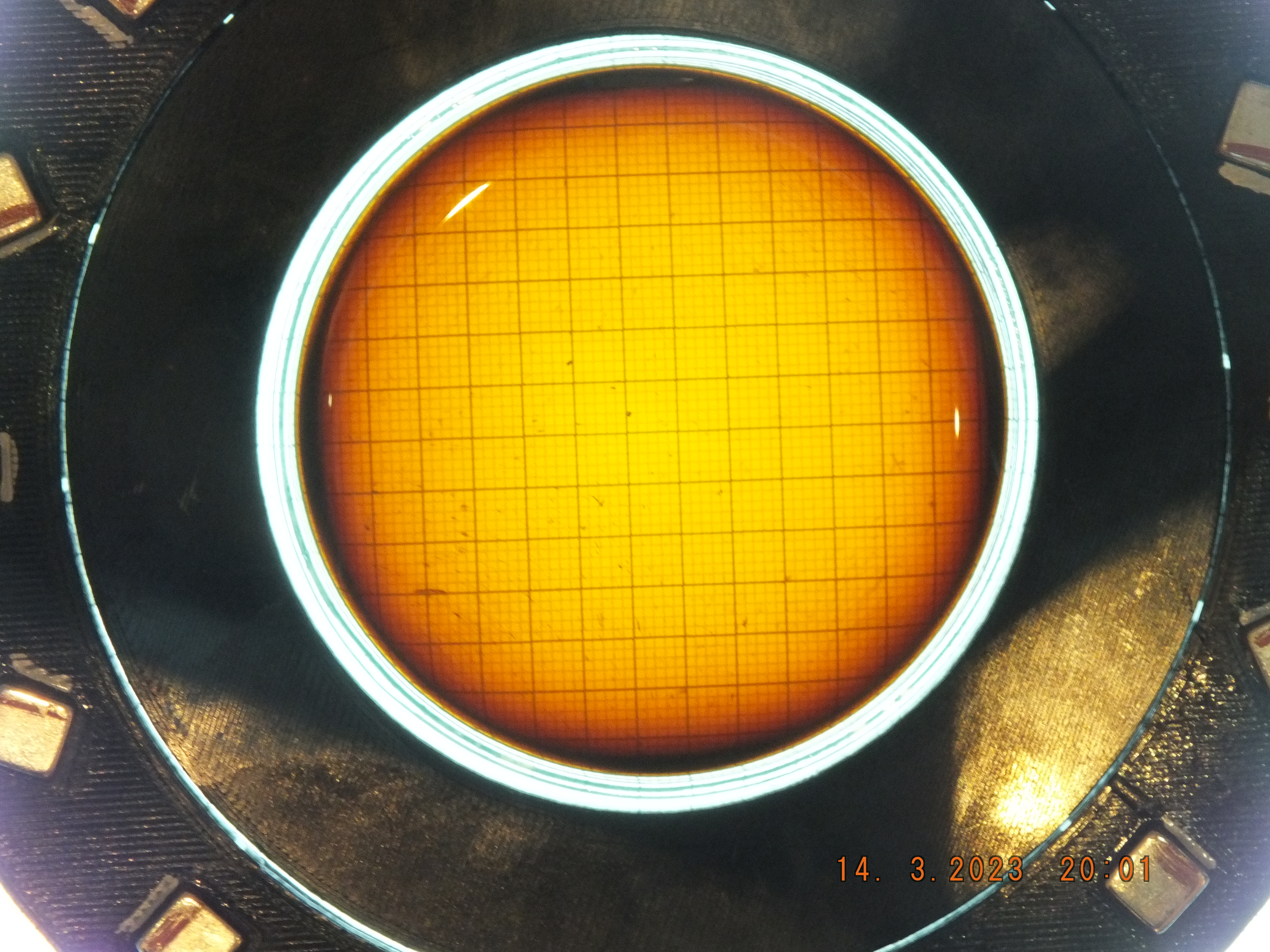

Supplement: Supplementary file 1 — Supplementary Information. [file 41598_2024_58091_MOESM1_ESM.zip › rawdata/fig7a/9_35.JPG]

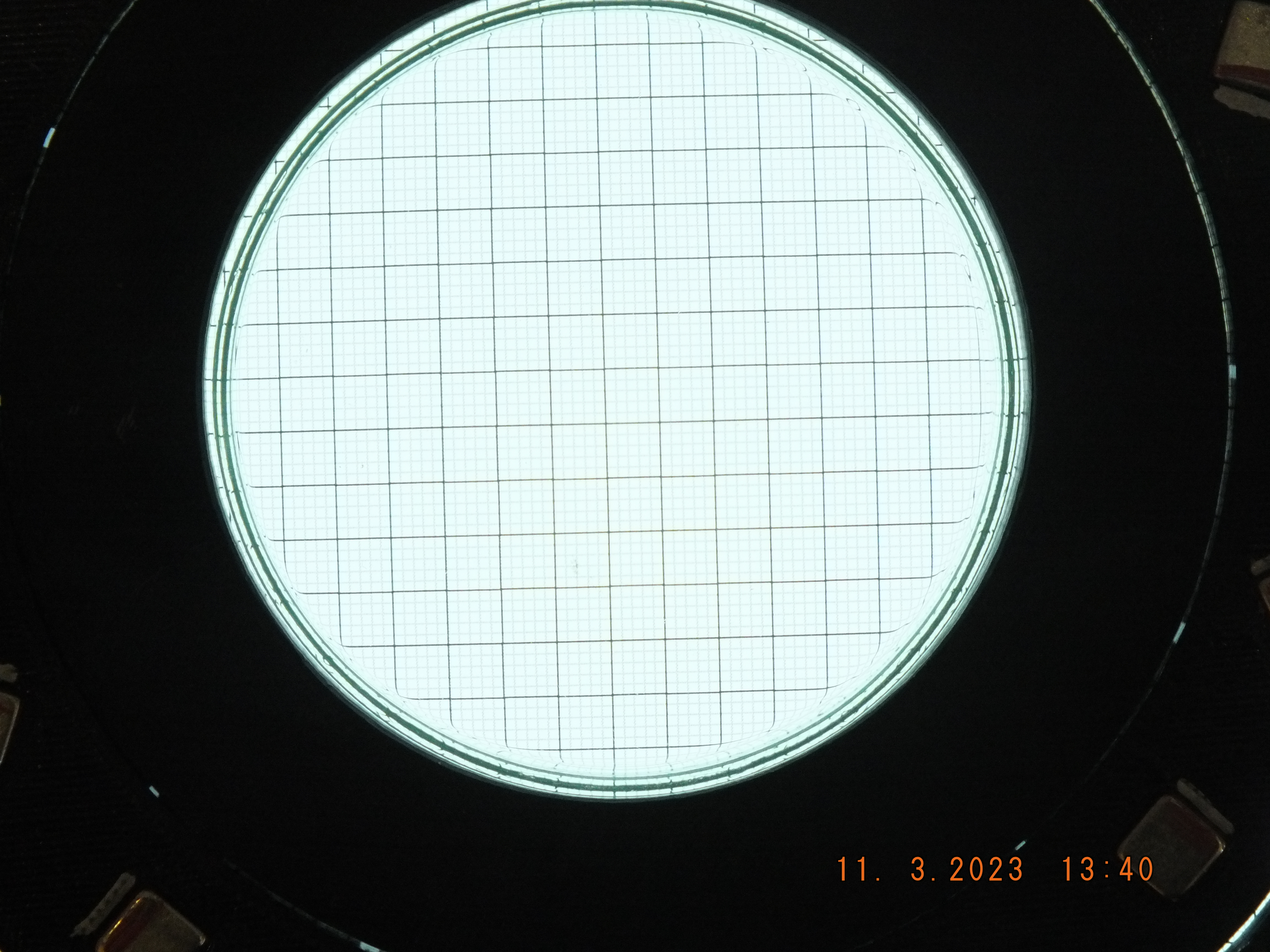

Supplement: Supplementary file 1 — Supplementary Information. [file 41598_2024_58091_MOESM1_ESM.zip › rawdata/fig7a/empty.JPG]

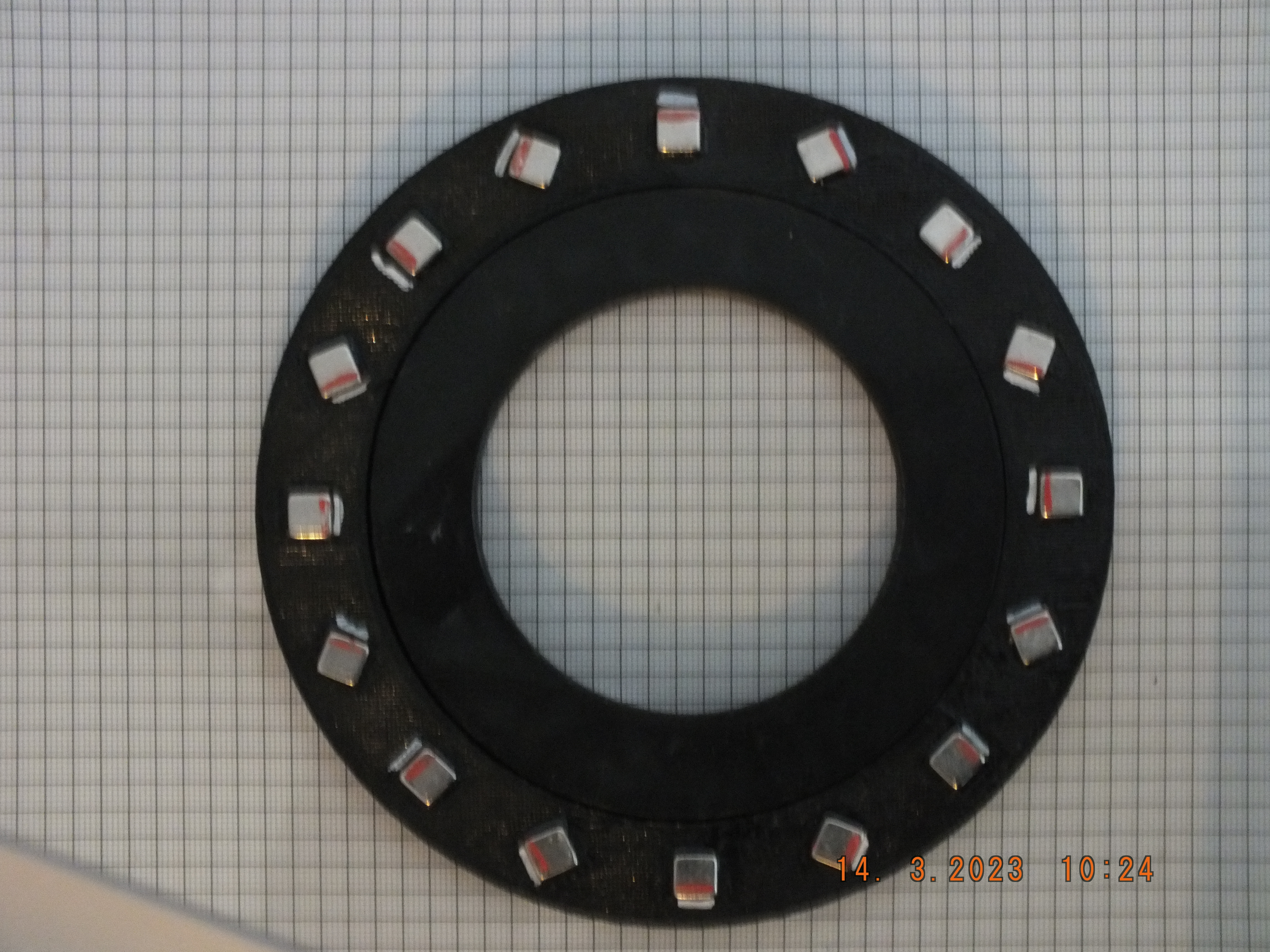

Supplement: Supplementary file 1 — Supplementary Information. [file 41598_2024_58091_MOESM1_ESM.zip › rawdata/fig7a/geometry.JPG]

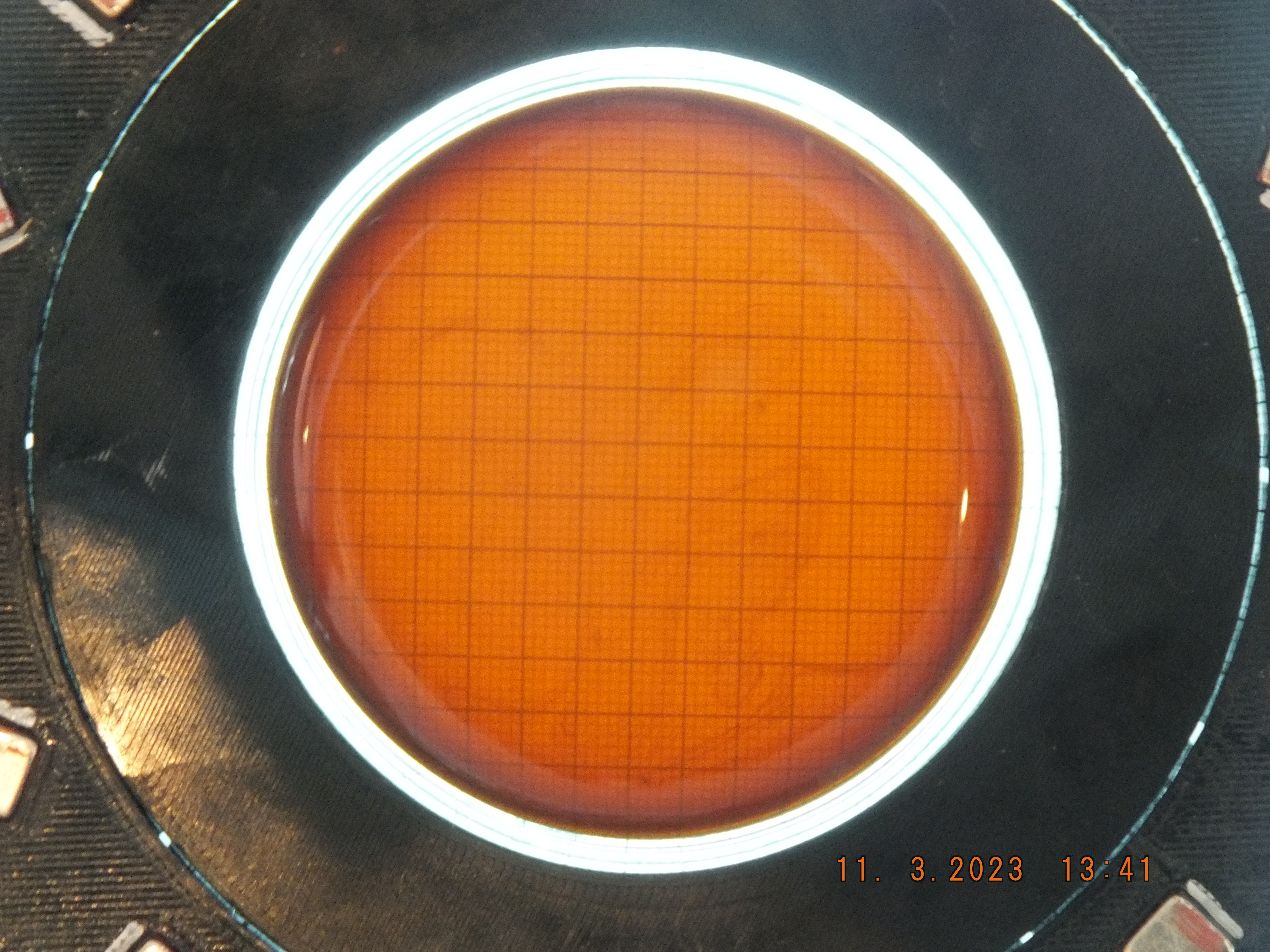

Supplement: Supplementary file 1 — Supplementary Information. [file 41598_2024_58091_MOESM1_ESM.zip › rawdata/fig7b/0.JPG]

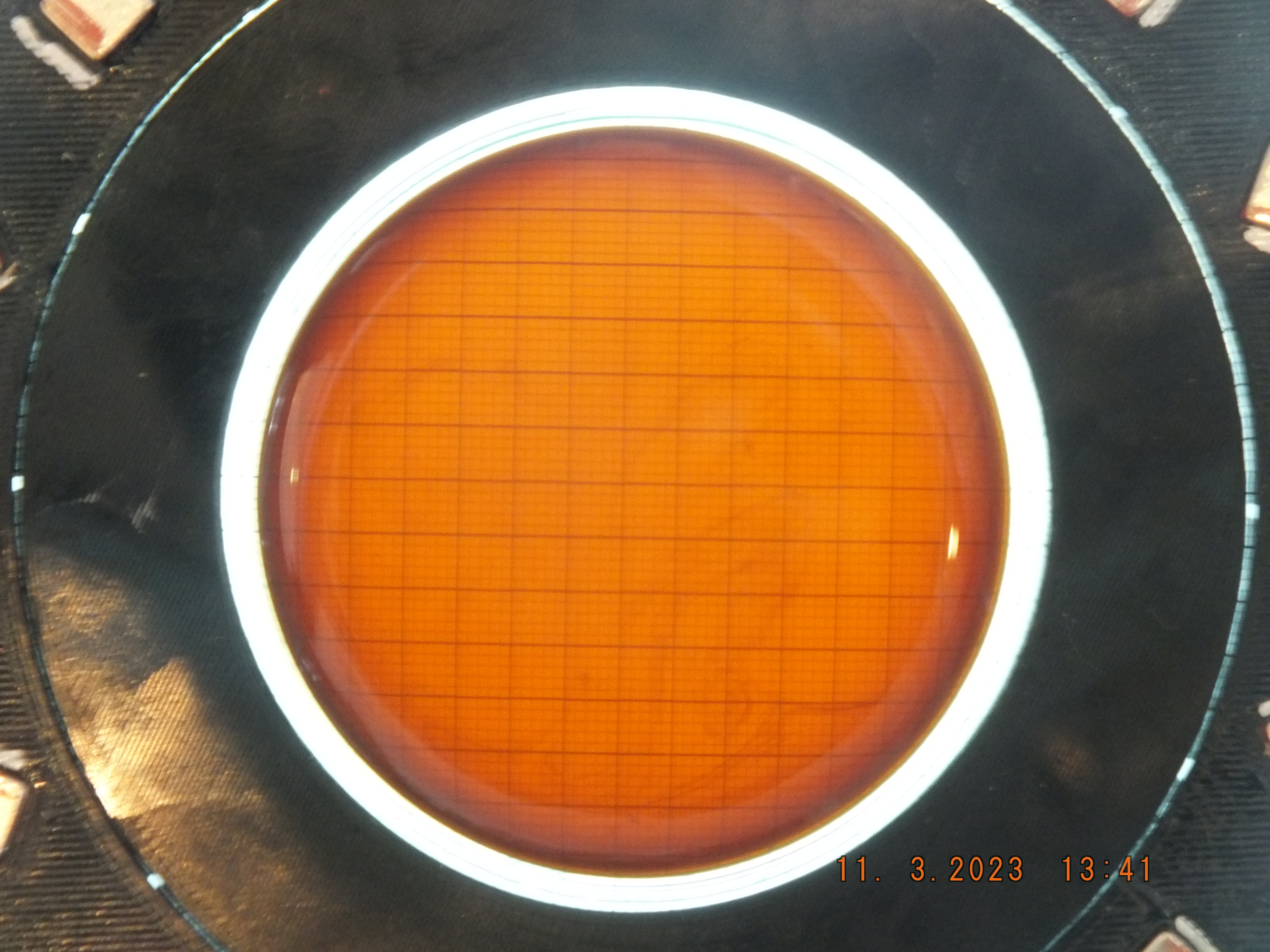

Supplement: Supplementary file 1 — Supplementary Information. [file 41598_2024_58091_MOESM1_ESM.zip › rawdata/fig7b/00a.JPG]

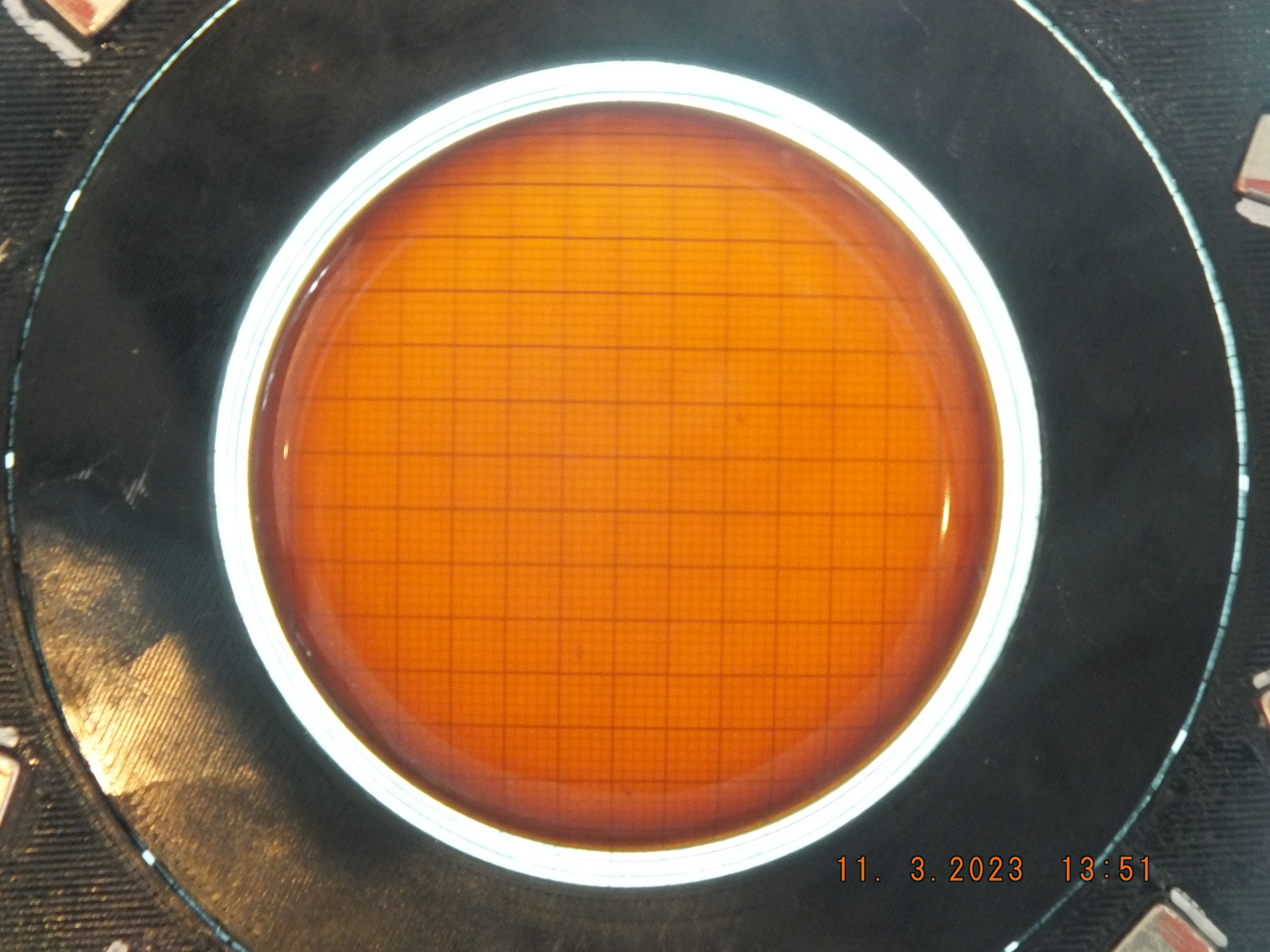

Supplement: Supplementary file 1 — Supplementary Information. [file 41598_2024_58091_MOESM1_ESM.zip › rawdata/fig7b/0_10.JPG]

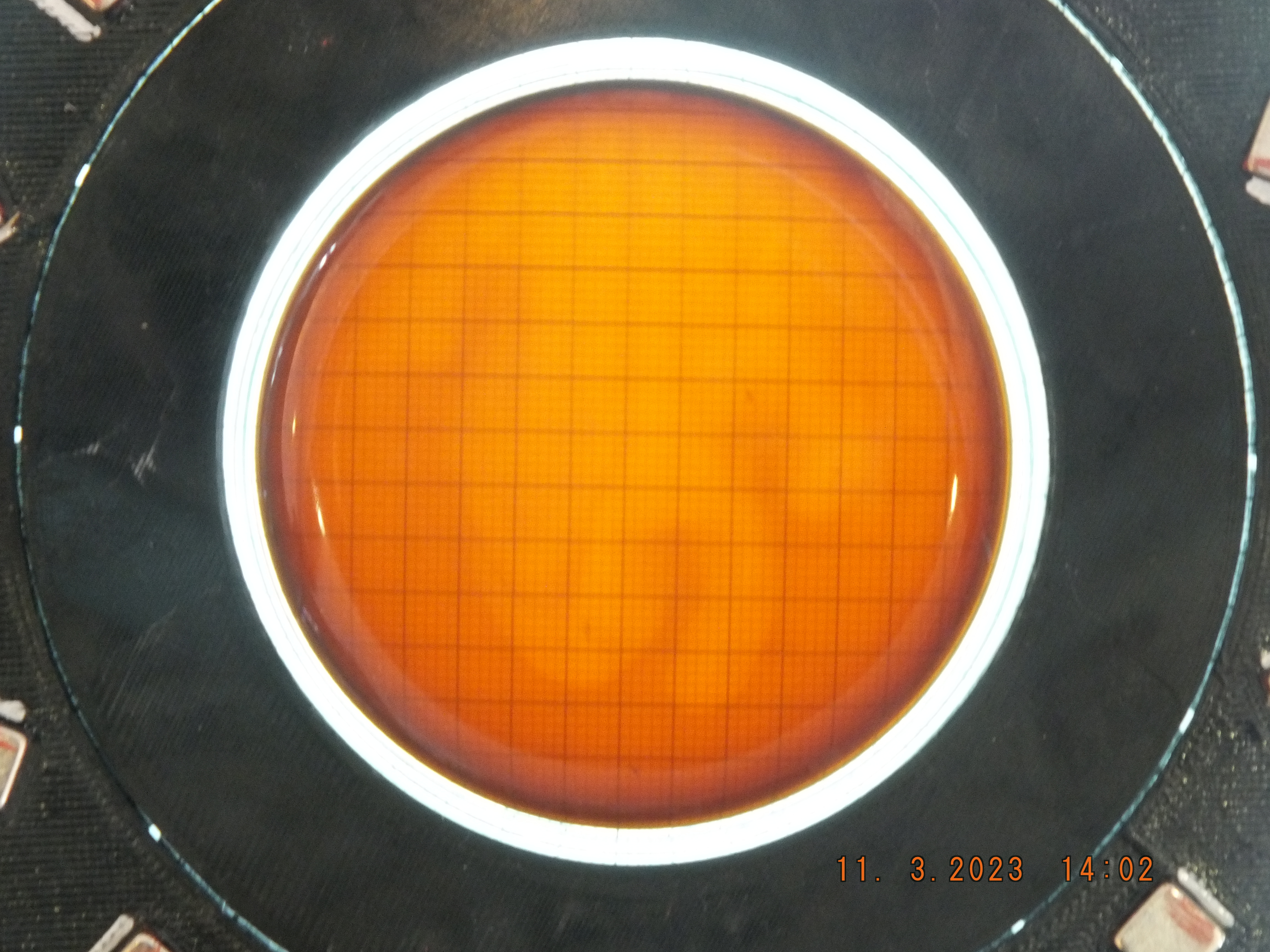

Supplement: Supplementary file 1 — Supplementary Information. [file 41598_2024_58091_MOESM1_ESM.zip › rawdata/fig7b/0_21.JPG]

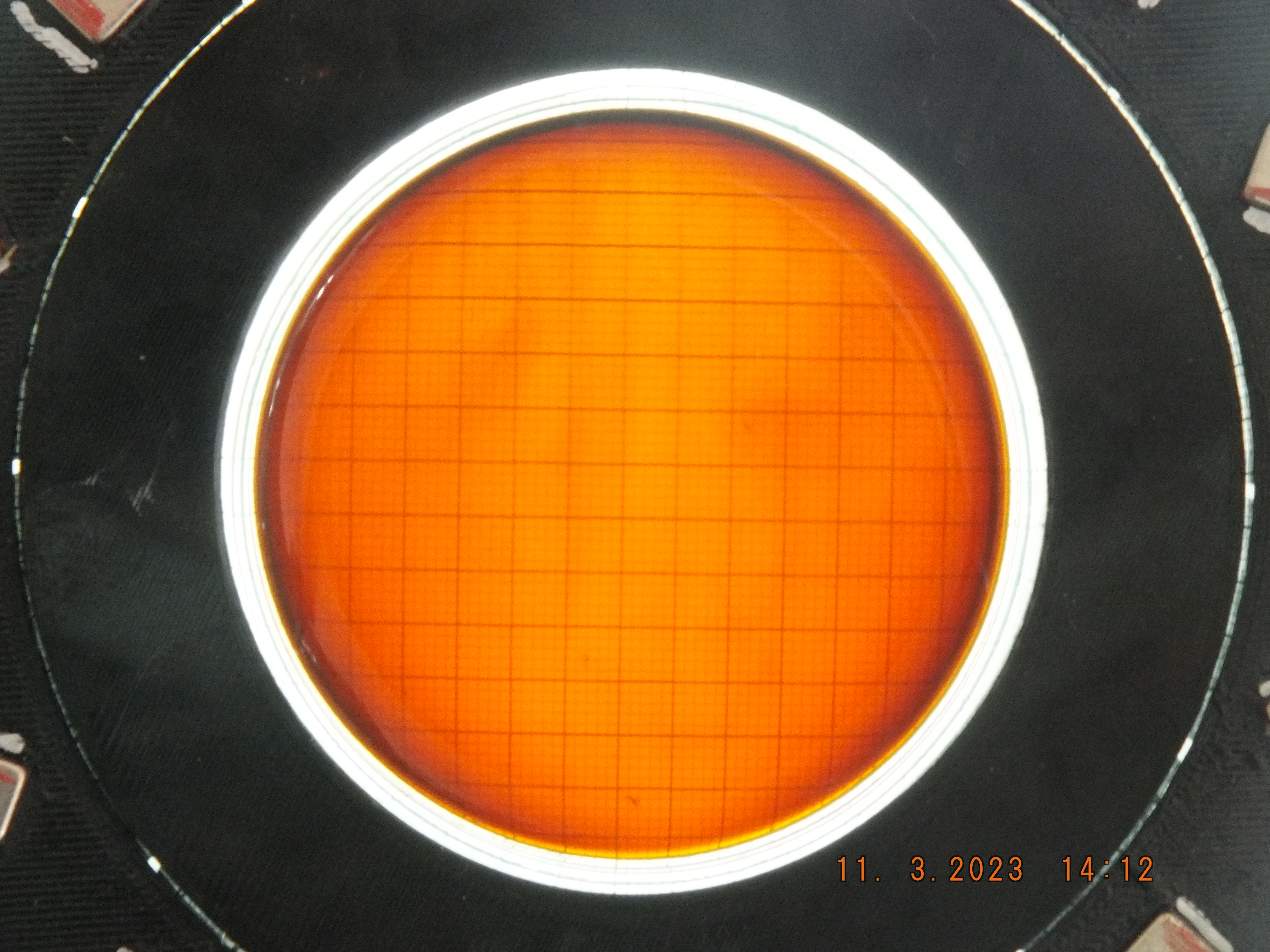

Supplement: Supplementary file 1 — Supplementary Information. [file 41598_2024_58091_MOESM1_ESM.zip › rawdata/fig7b/0_31.JPG]

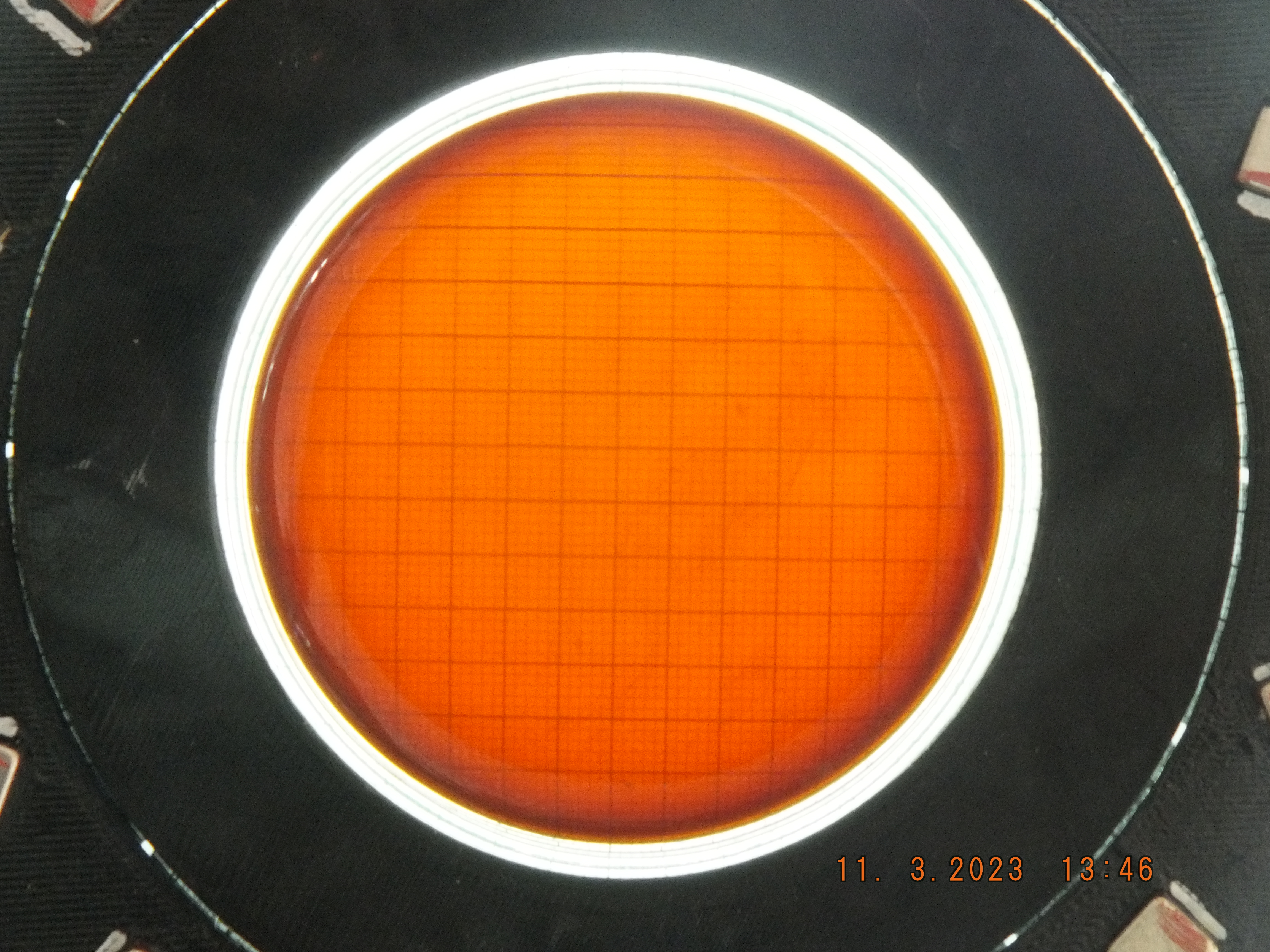

Supplement: Supplementary file 1 — Supplementary Information. [file 41598_2024_58091_MOESM1_ESM.zip › rawdata/fig7b/0_5.JPG]

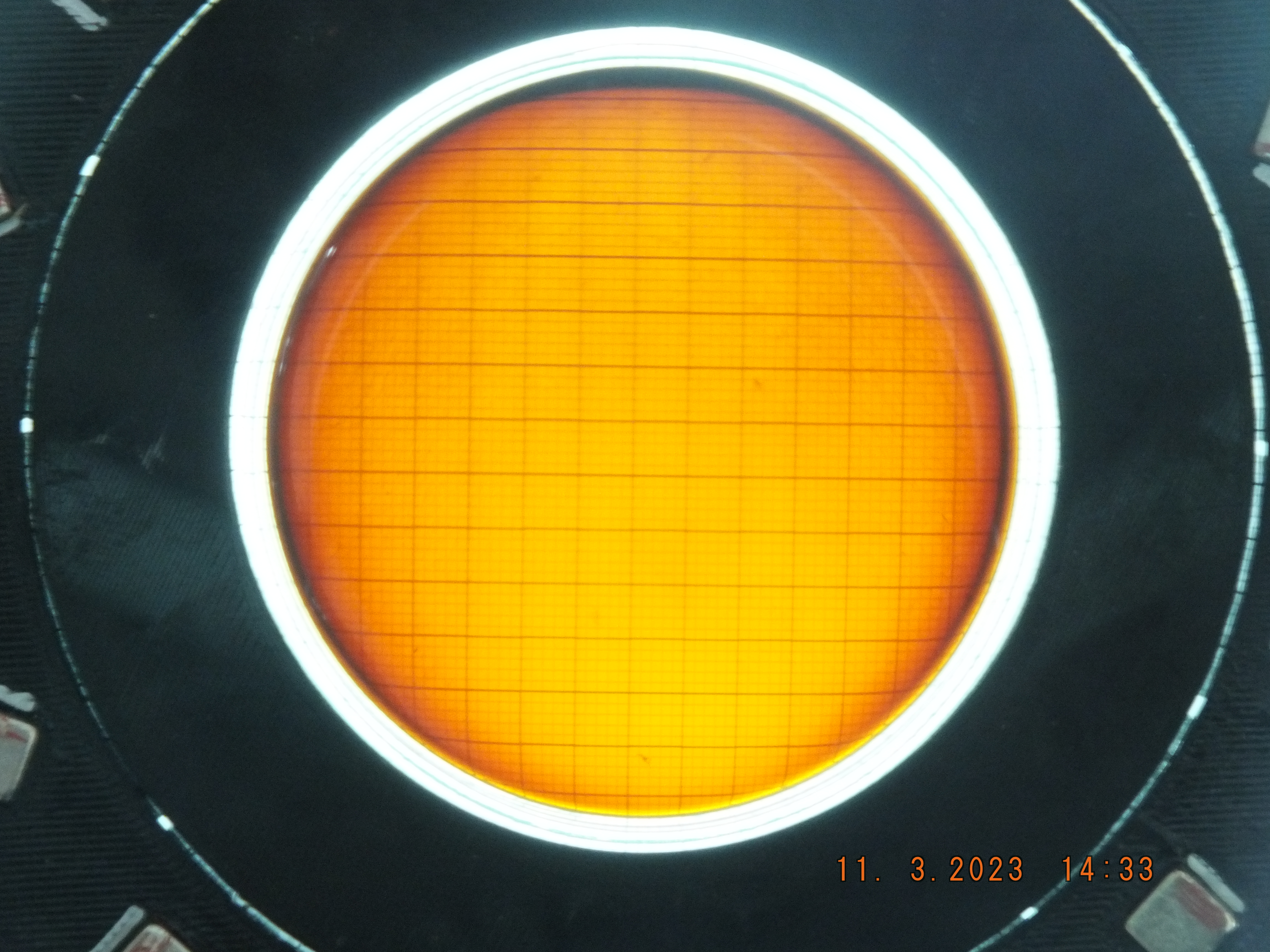

Supplement: Supplementary file 1 — Supplementary Information. [file 41598_2024_58091_MOESM1_ESM.zip › rawdata/fig7b/0_52.JPG]

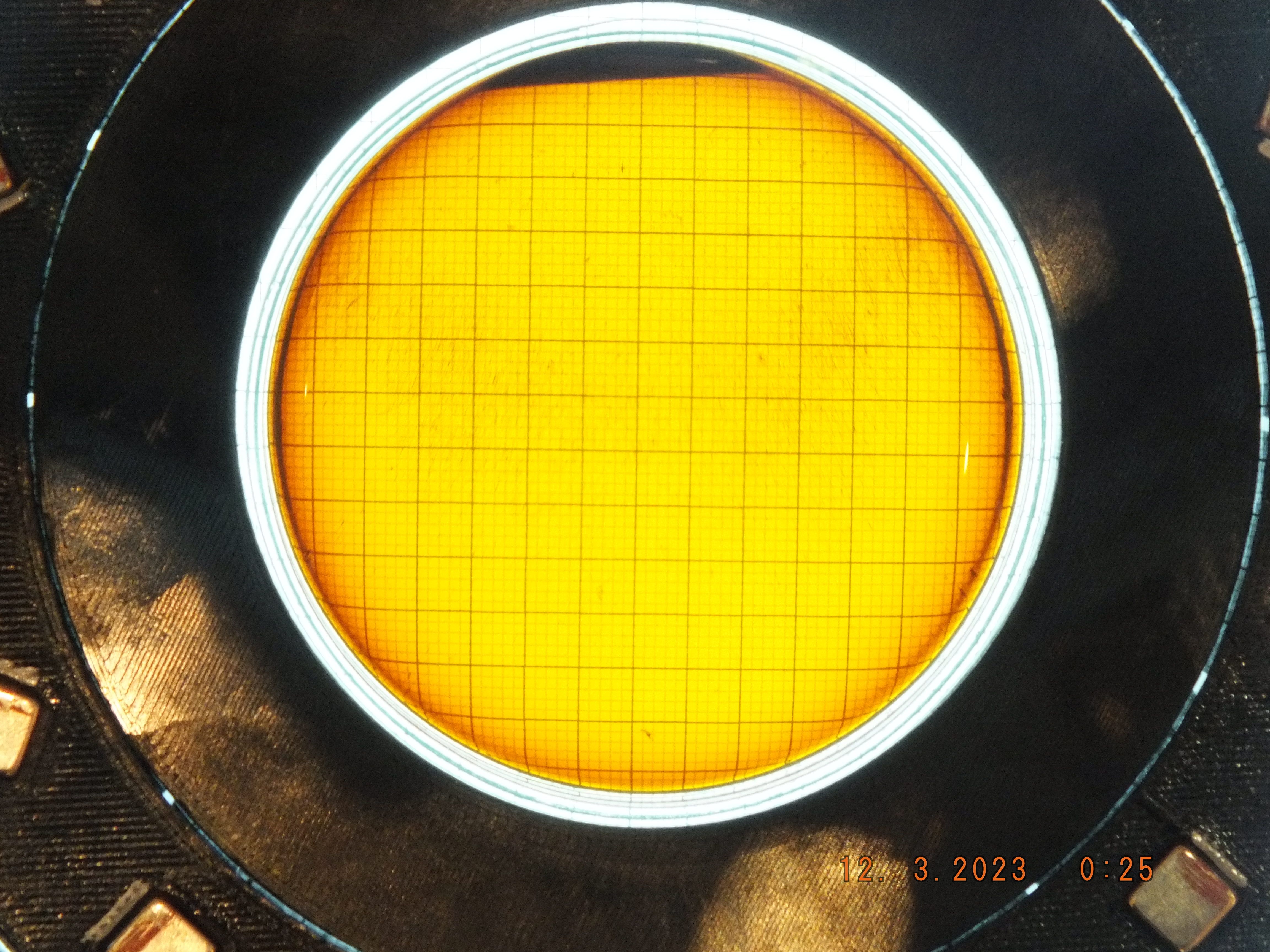

Supplement: Supplementary file 1 — Supplementary Information. [file 41598_2024_58091_MOESM1_ESM.zip › rawdata/fig7b/10_44.JPG]

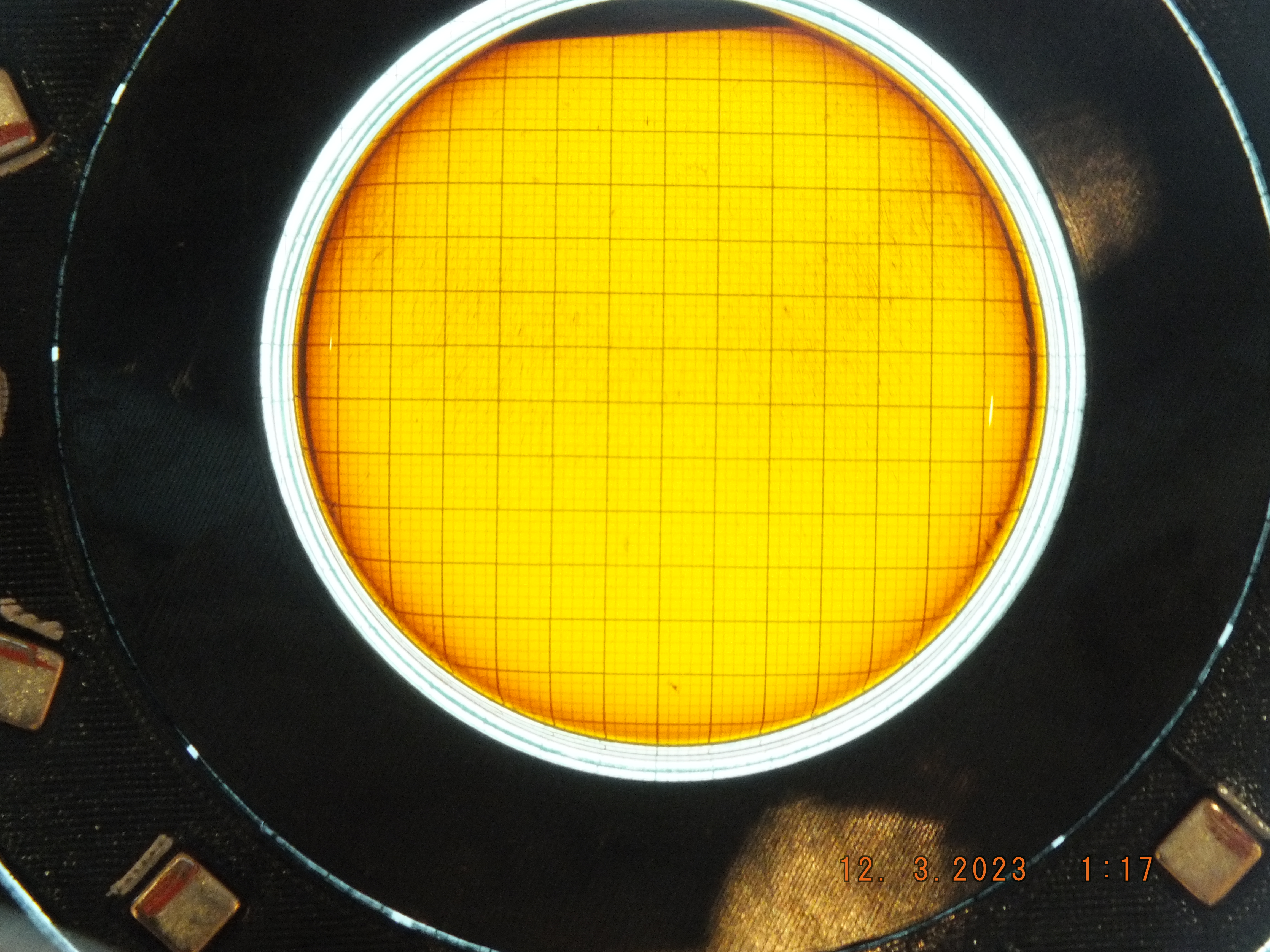

Supplement: Supplementary file 1 — Supplementary Information. [file 41598_2024_58091_MOESM1_ESM.zip › rawdata/fig7b/11_36.JPG]

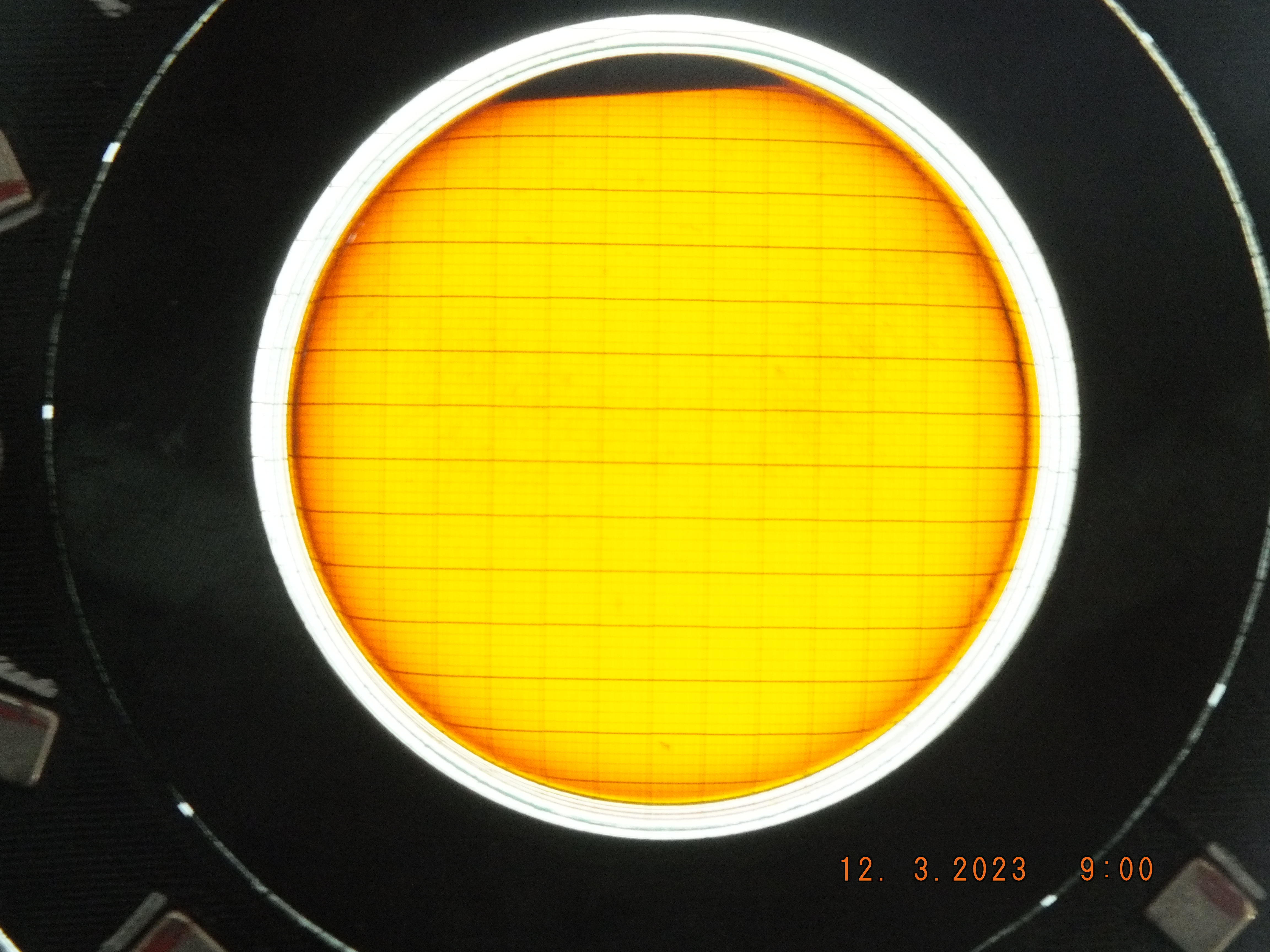

Supplement: Supplementary file 1 — Supplementary Information. [file 41598_2024_58091_MOESM1_ESM.zip › rawdata/fig7b/19_19.JPG]

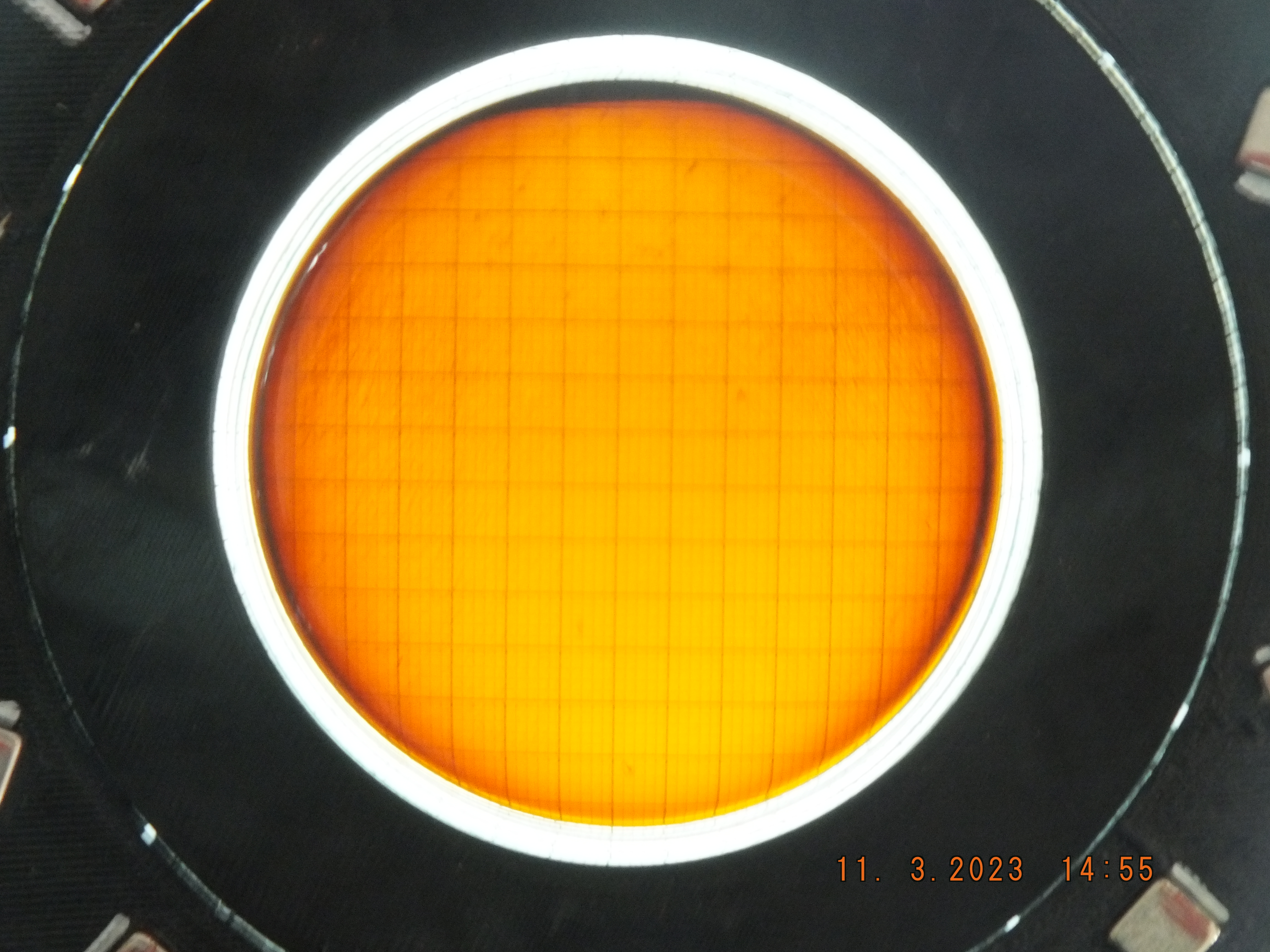

Supplement: Supplementary file 1 — Supplementary Information. [file 41598_2024_58091_MOESM1_ESM.zip › rawdata/fig7b/1_14.JPG]

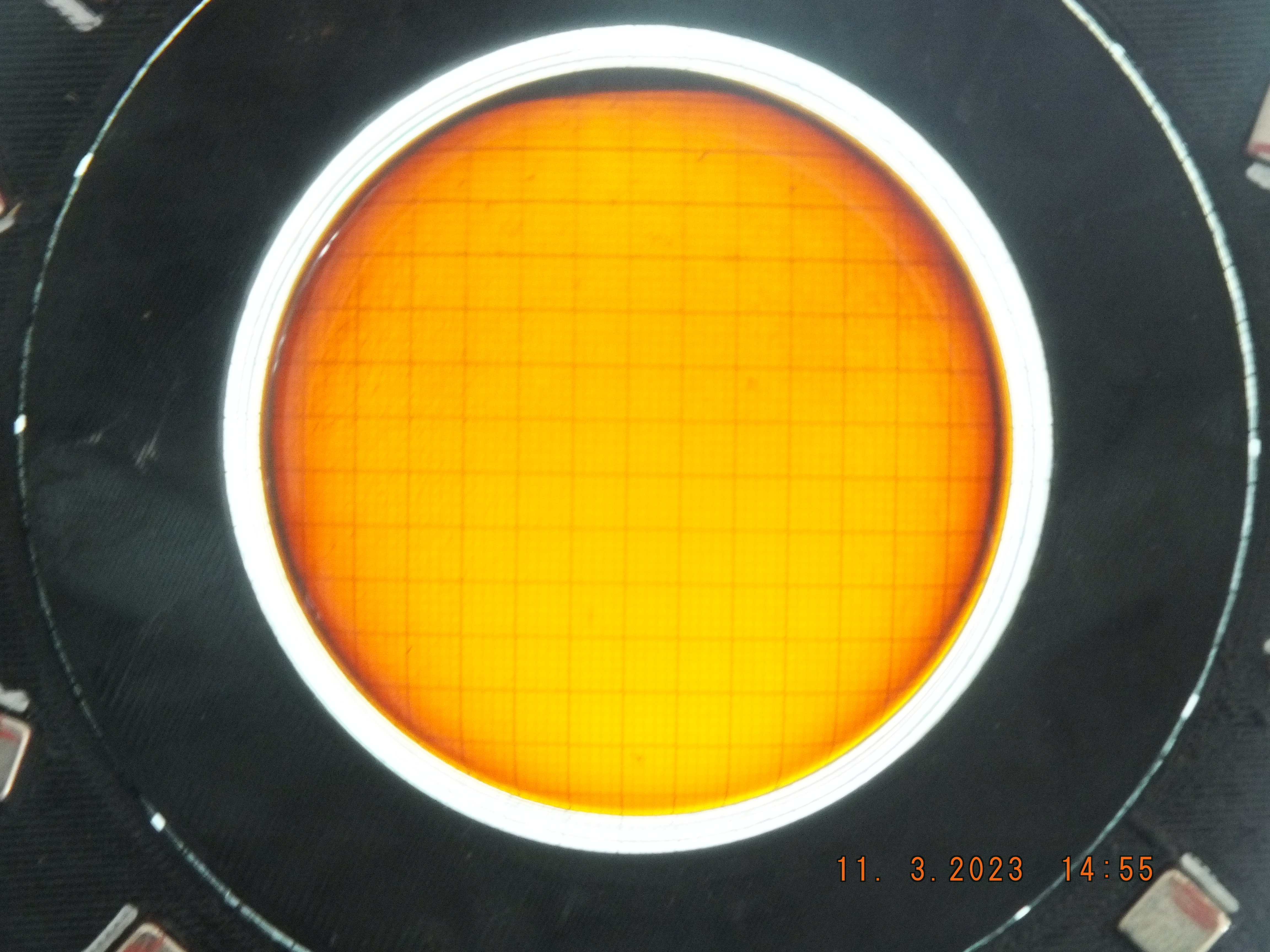

Supplement: Supplementary file 1 — Supplementary Information. [file 41598_2024_58091_MOESM1_ESM.zip › rawdata/fig7b/1_14a.JPG]

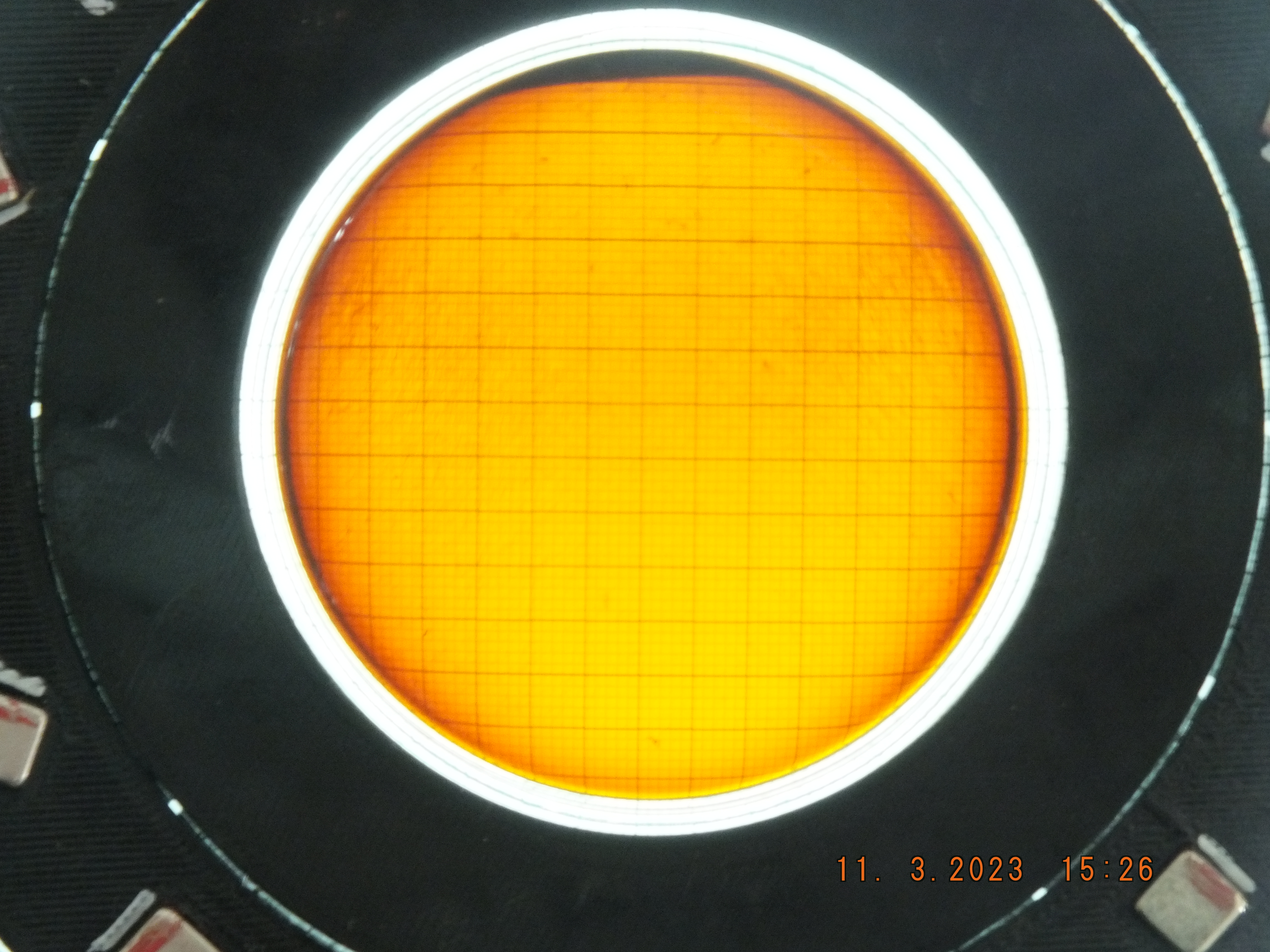

Supplement: Supplementary file 1 — Supplementary Information. [file 41598_2024_58091_MOESM1_ESM.zip › rawdata/fig7b/1_45.JPG]

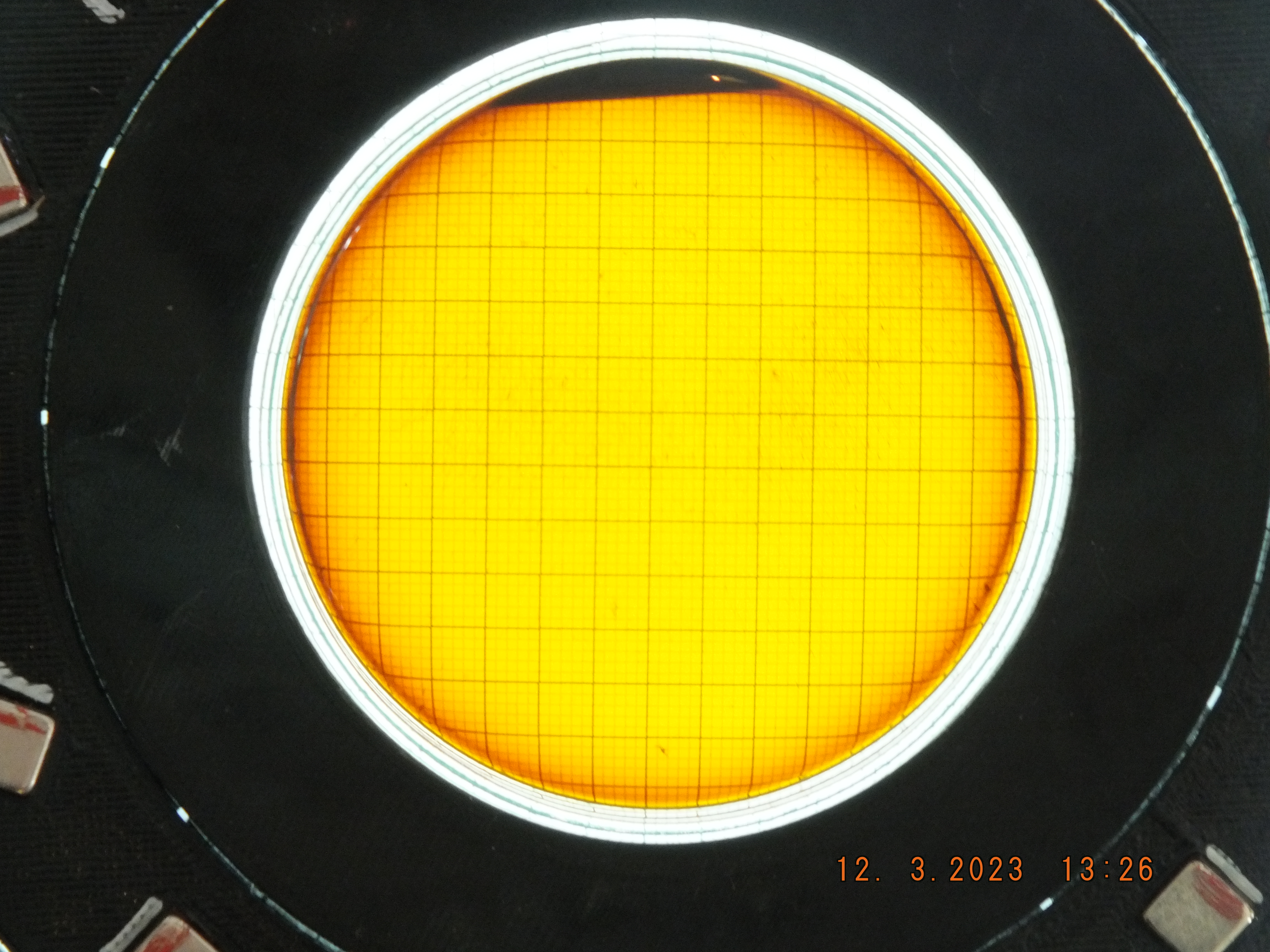

Supplement: Supplementary file 1 — Supplementary Information. [file 41598_2024_58091_MOESM1_ESM.zip › rawdata/fig7b/23_45.JPG]

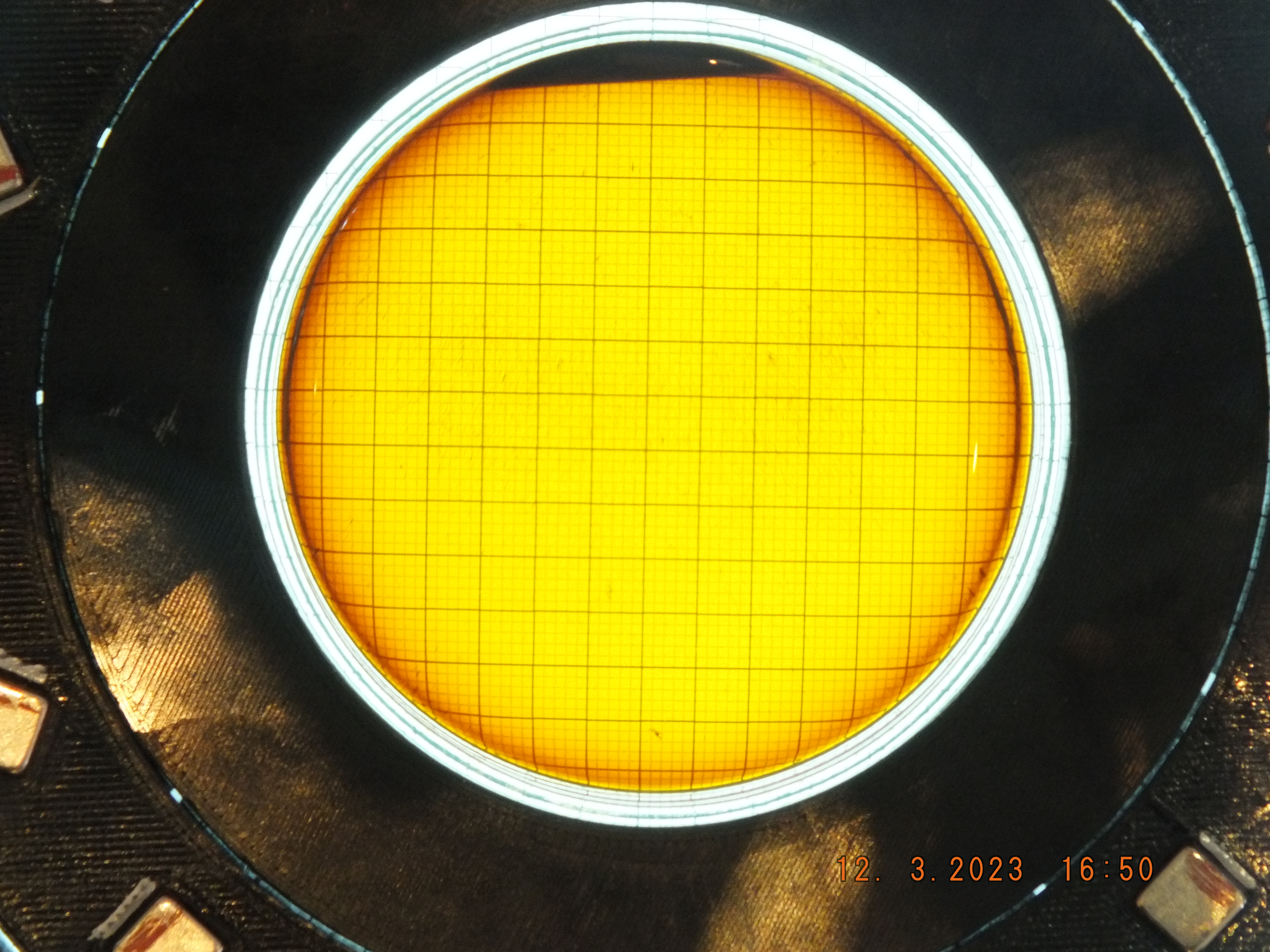

Supplement: Supplementary file 1 — Supplementary Information. [file 41598_2024_58091_MOESM1_ESM.zip › rawdata/fig7b/27_09.JPG]

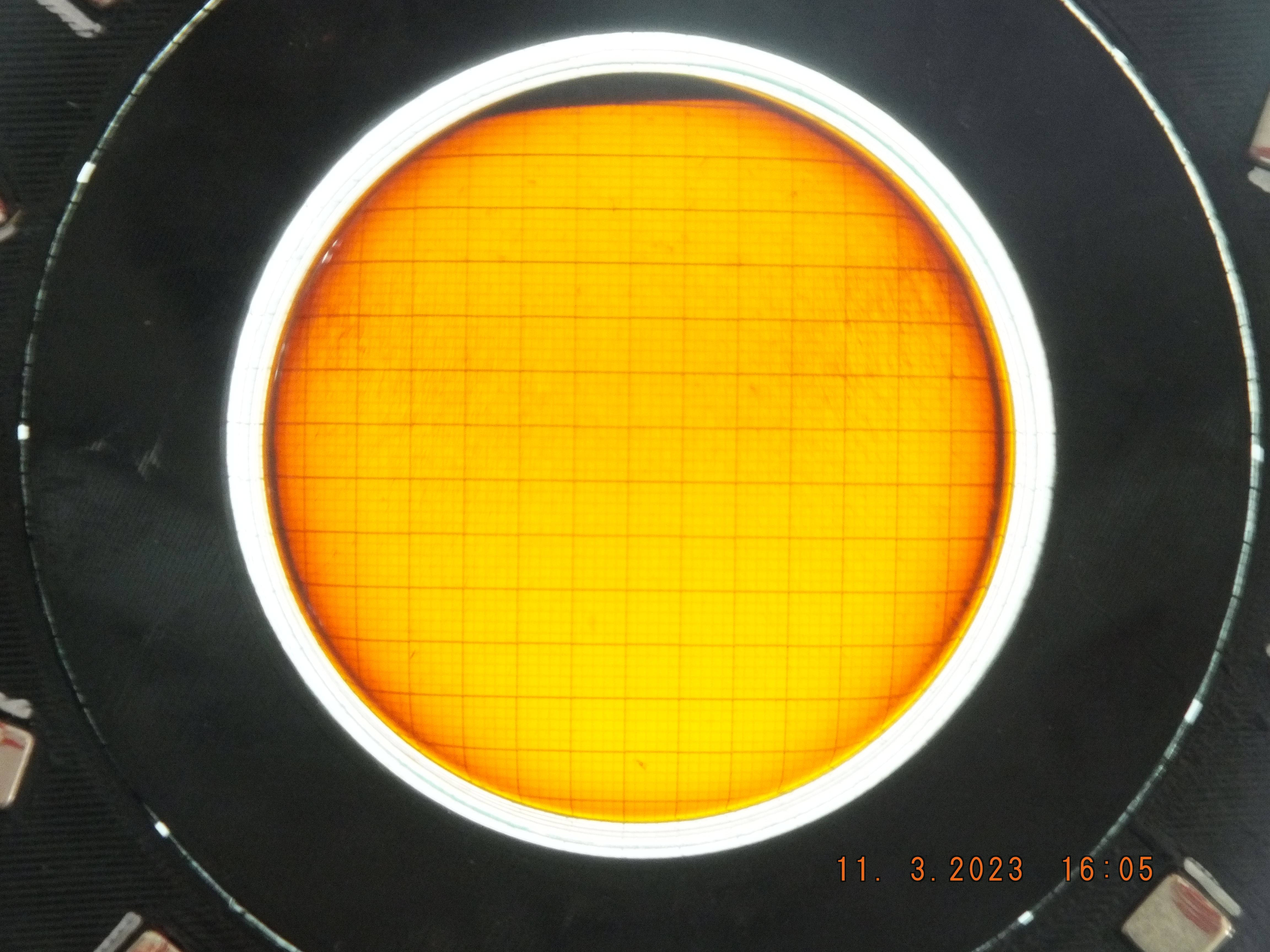

Supplement: Supplementary file 1 — Supplementary Information. [file 41598_2024_58091_MOESM1_ESM.zip › rawdata/fig7b/2_24.JPG]

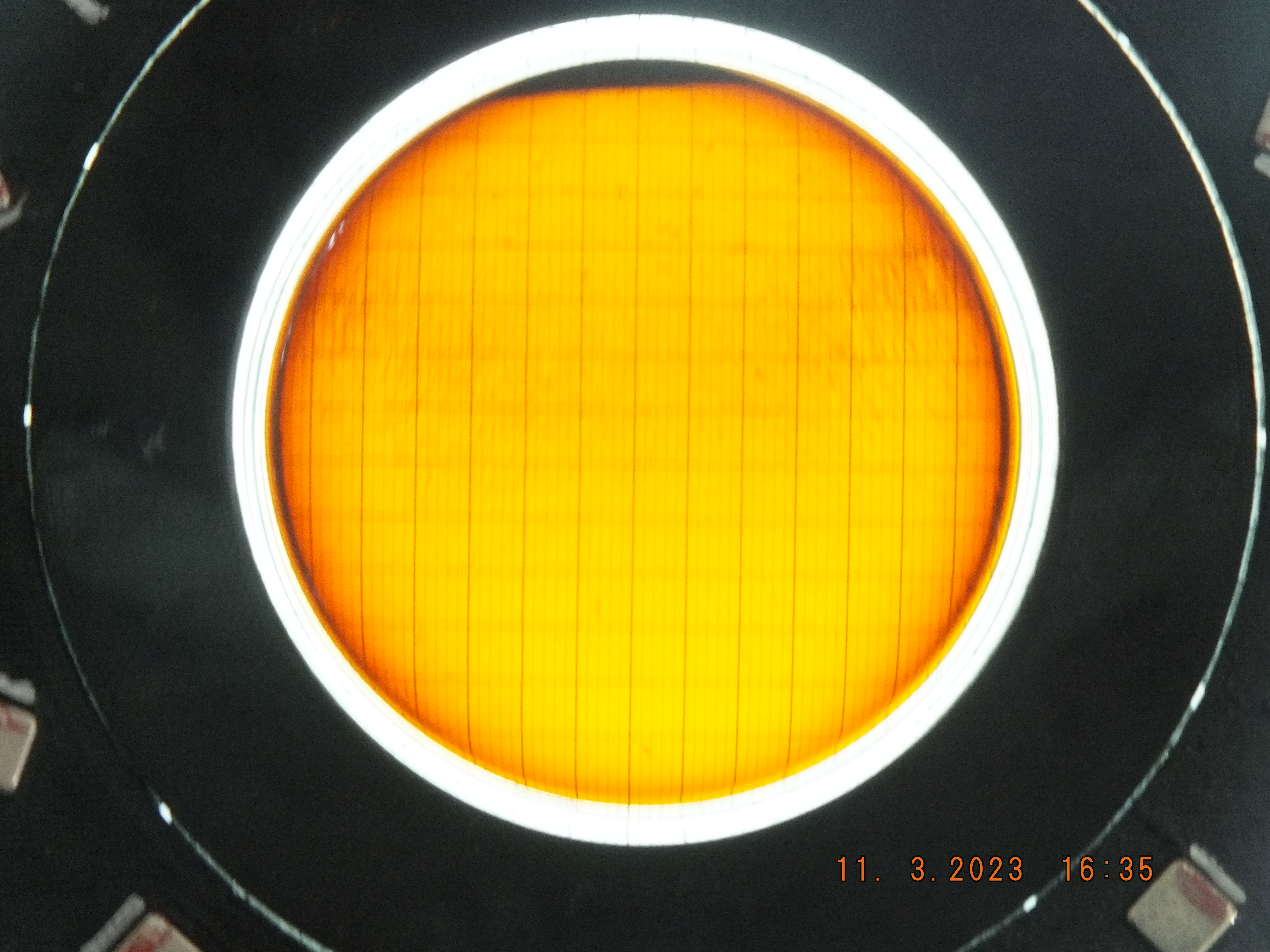

Supplement: Supplementary file 1 — Supplementary Information. [file 41598_2024_58091_MOESM1_ESM.zip › rawdata/fig7b/2_54.JPG]

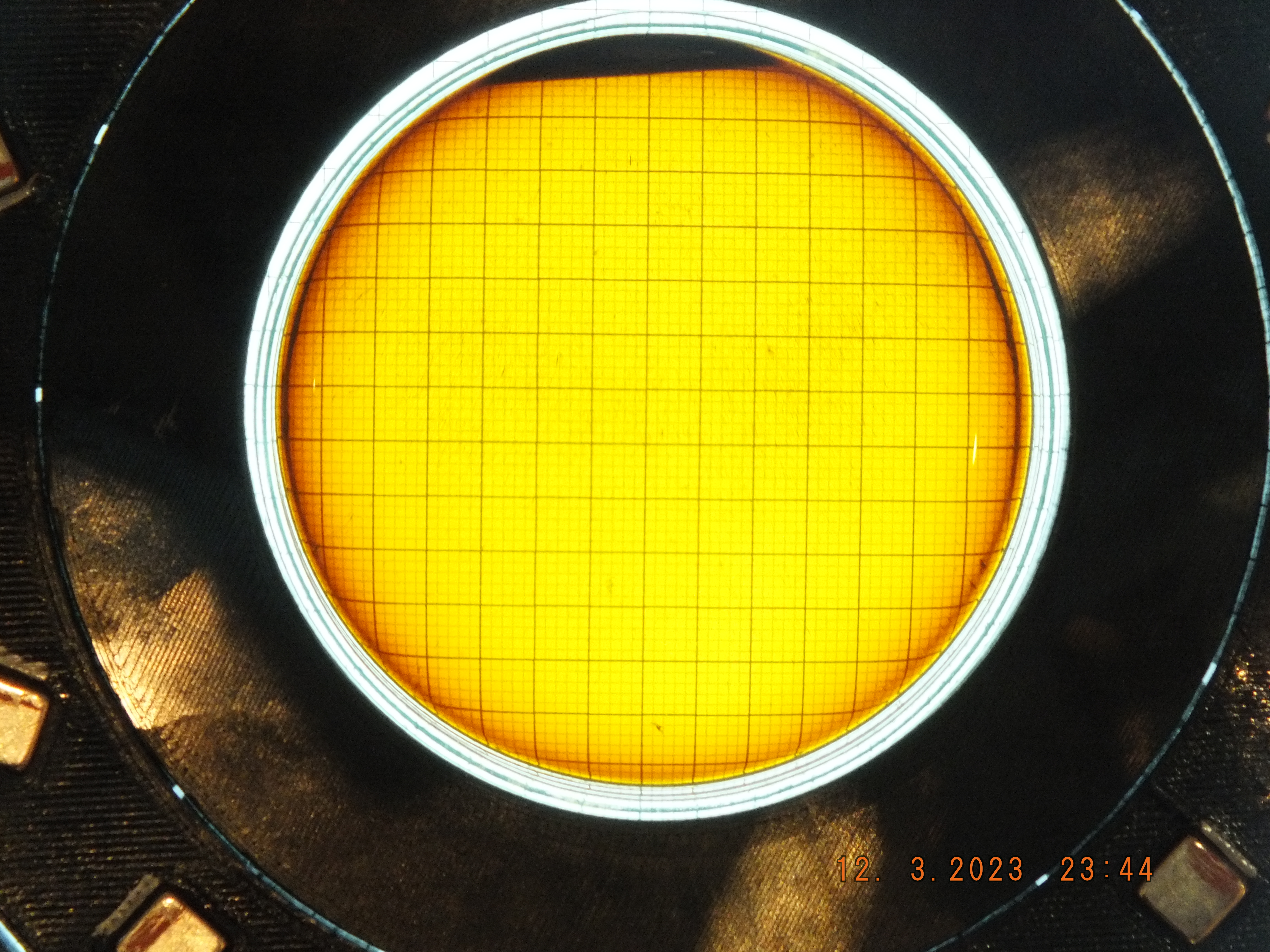

Supplement: Supplementary file 1 — Supplementary Information. [file 41598_2024_58091_MOESM1_ESM.zip › rawdata/fig7b/34_03.JPG]

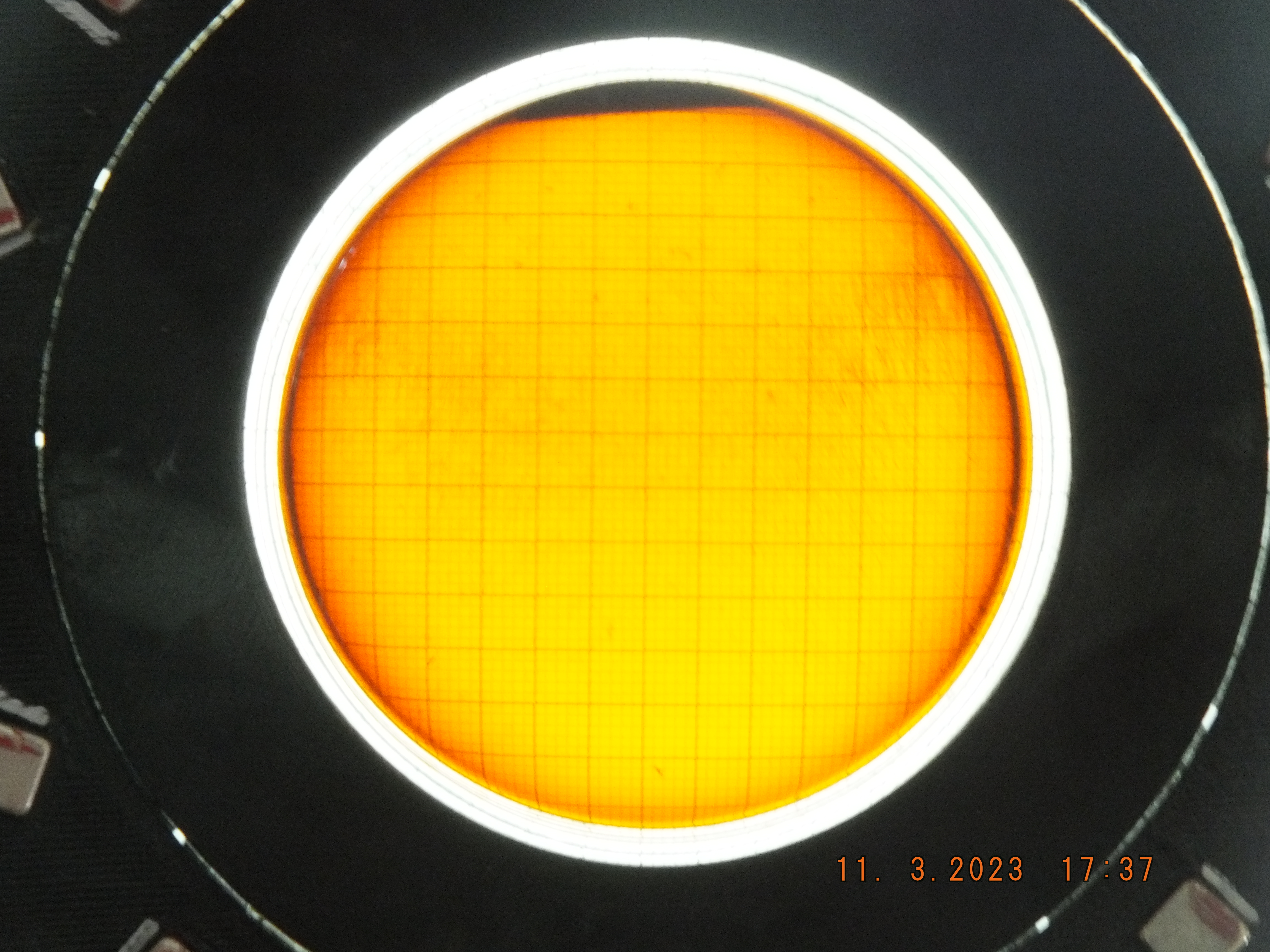

Supplement: Supplementary file 1 — Supplementary Information. [file 41598_2024_58091_MOESM1_ESM.zip › rawdata/fig7b/3_56.JPG]

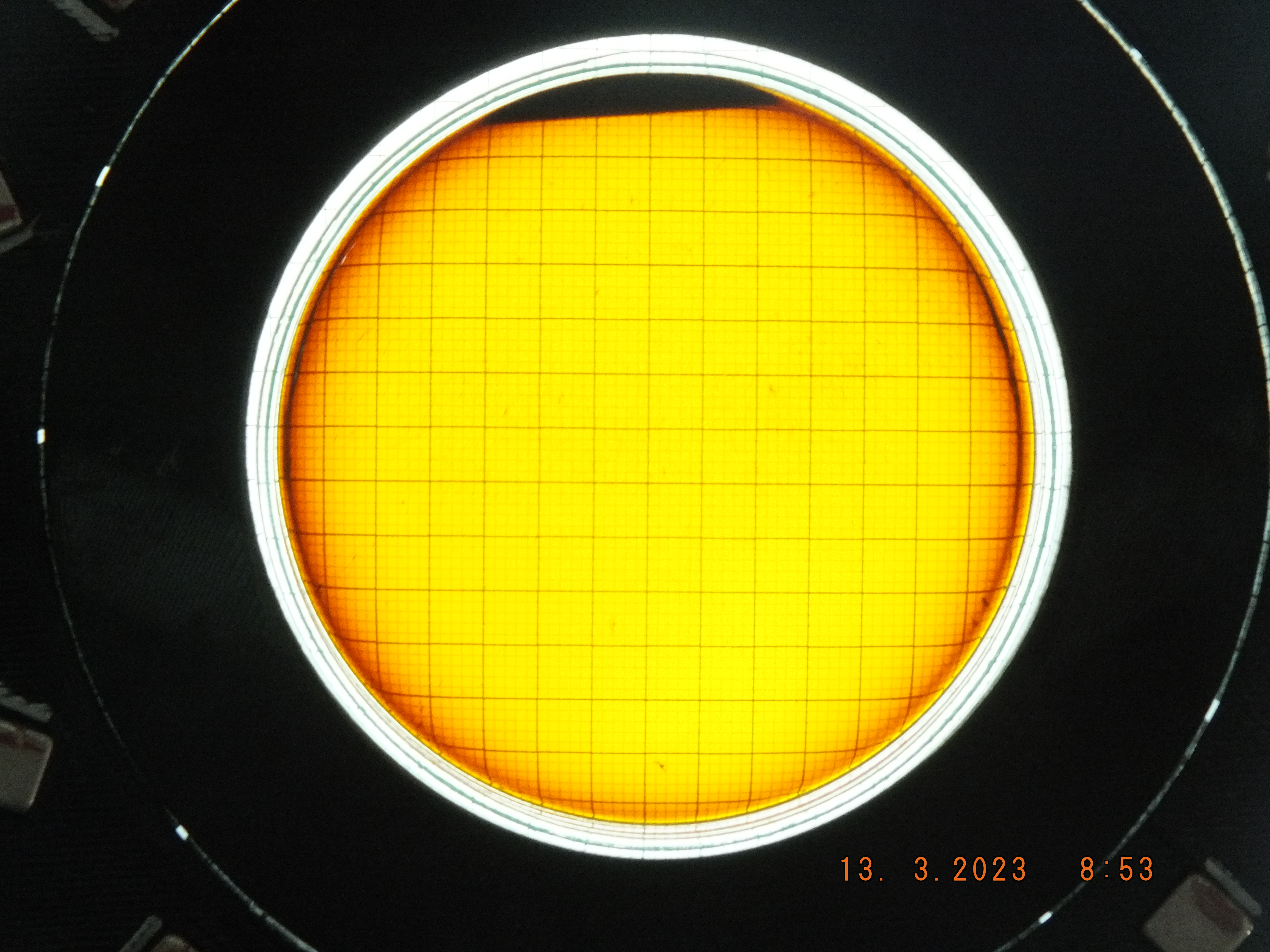

Supplement: Supplementary file 1 — Supplementary Information. [file 41598_2024_58091_MOESM1_ESM.zip › rawdata/fig7b/43_12.JPG]

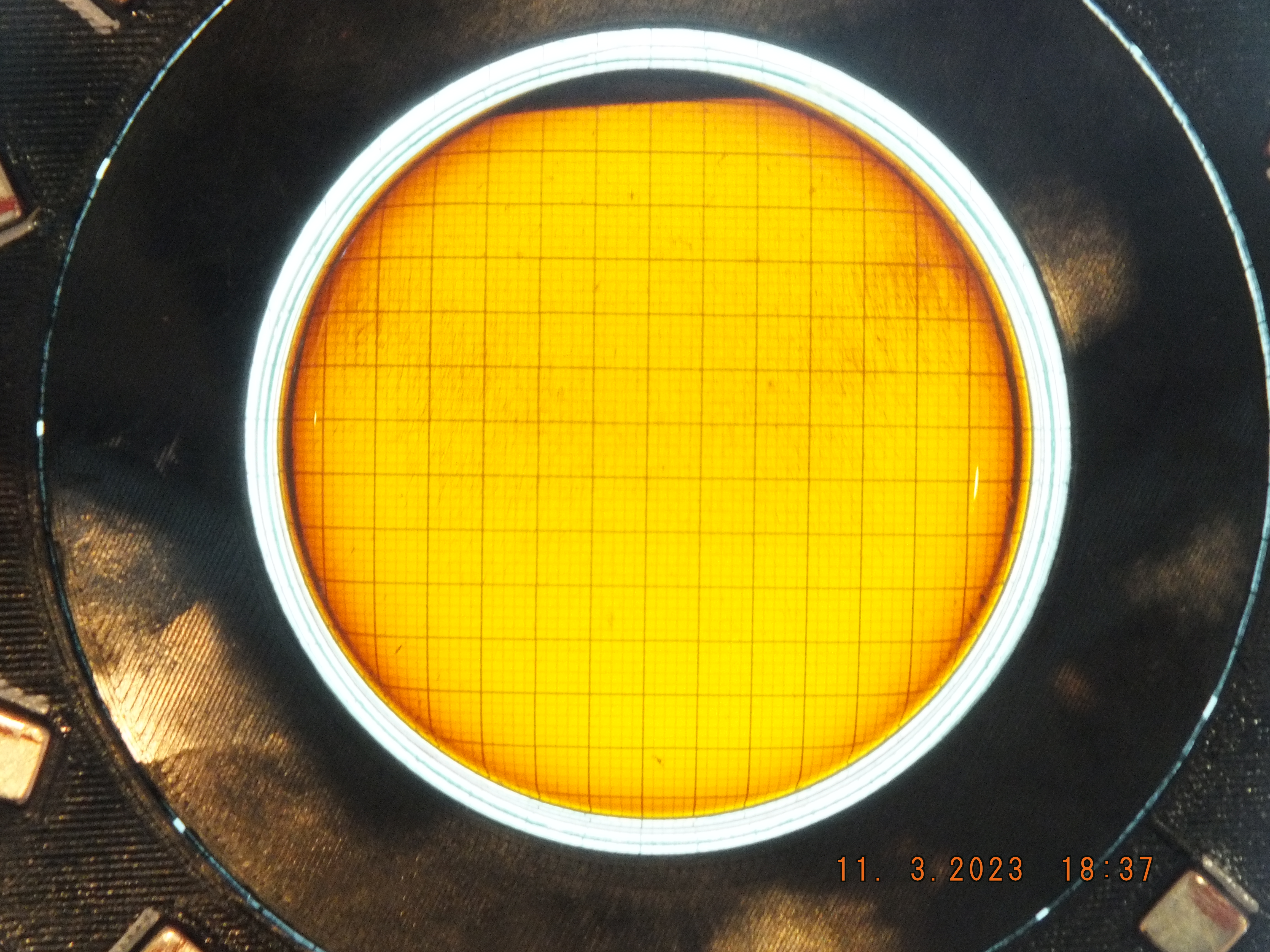

Supplement: Supplementary file 1 — Supplementary Information. [file 41598_2024_58091_MOESM1_ESM.zip › rawdata/fig7b/4_56.JPG]

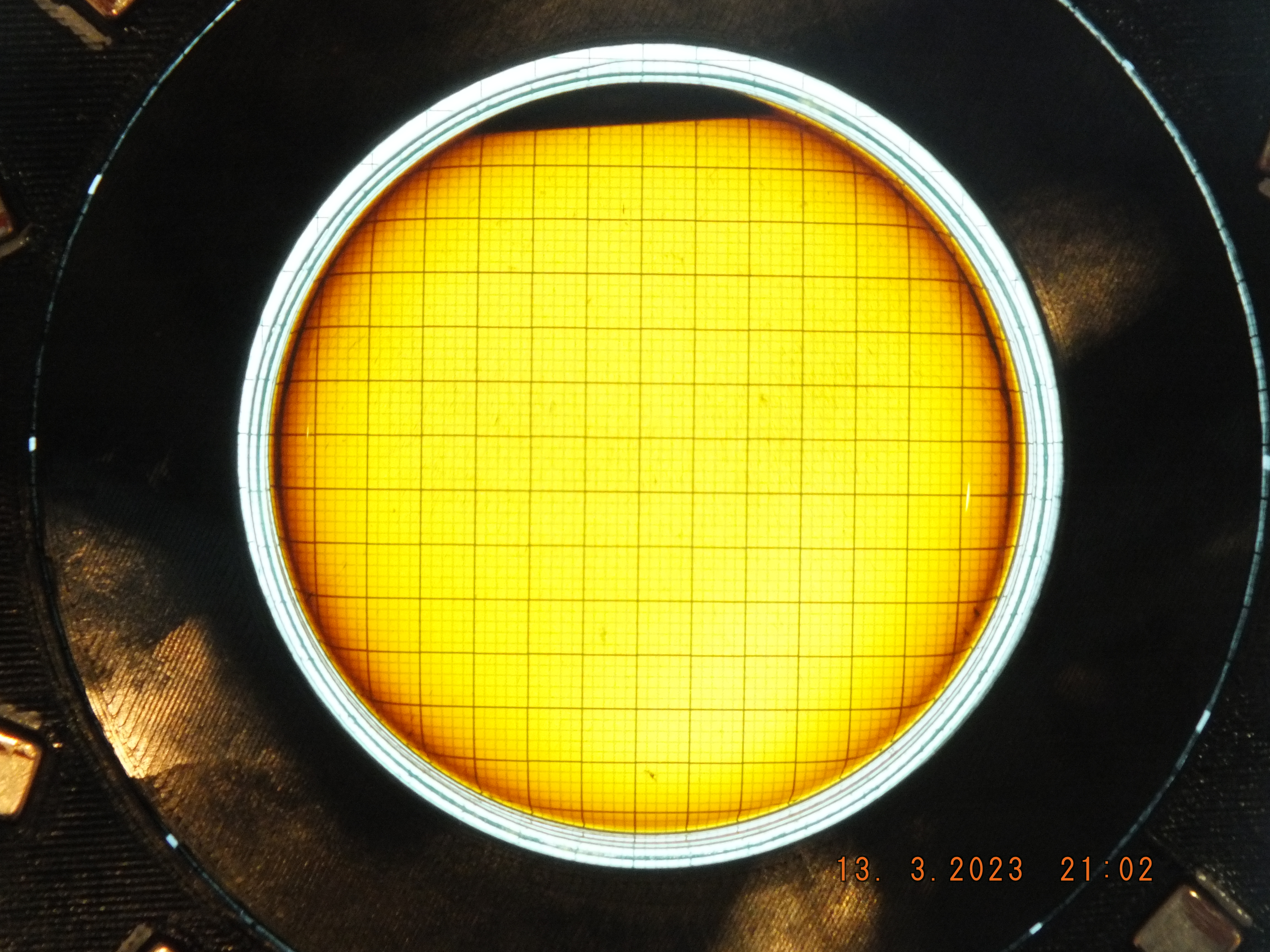

Supplement: Supplementary file 1 — Supplementary Information. [file 41598_2024_58091_MOESM1_ESM.zip › rawdata/fig7b/55_21.JPG]

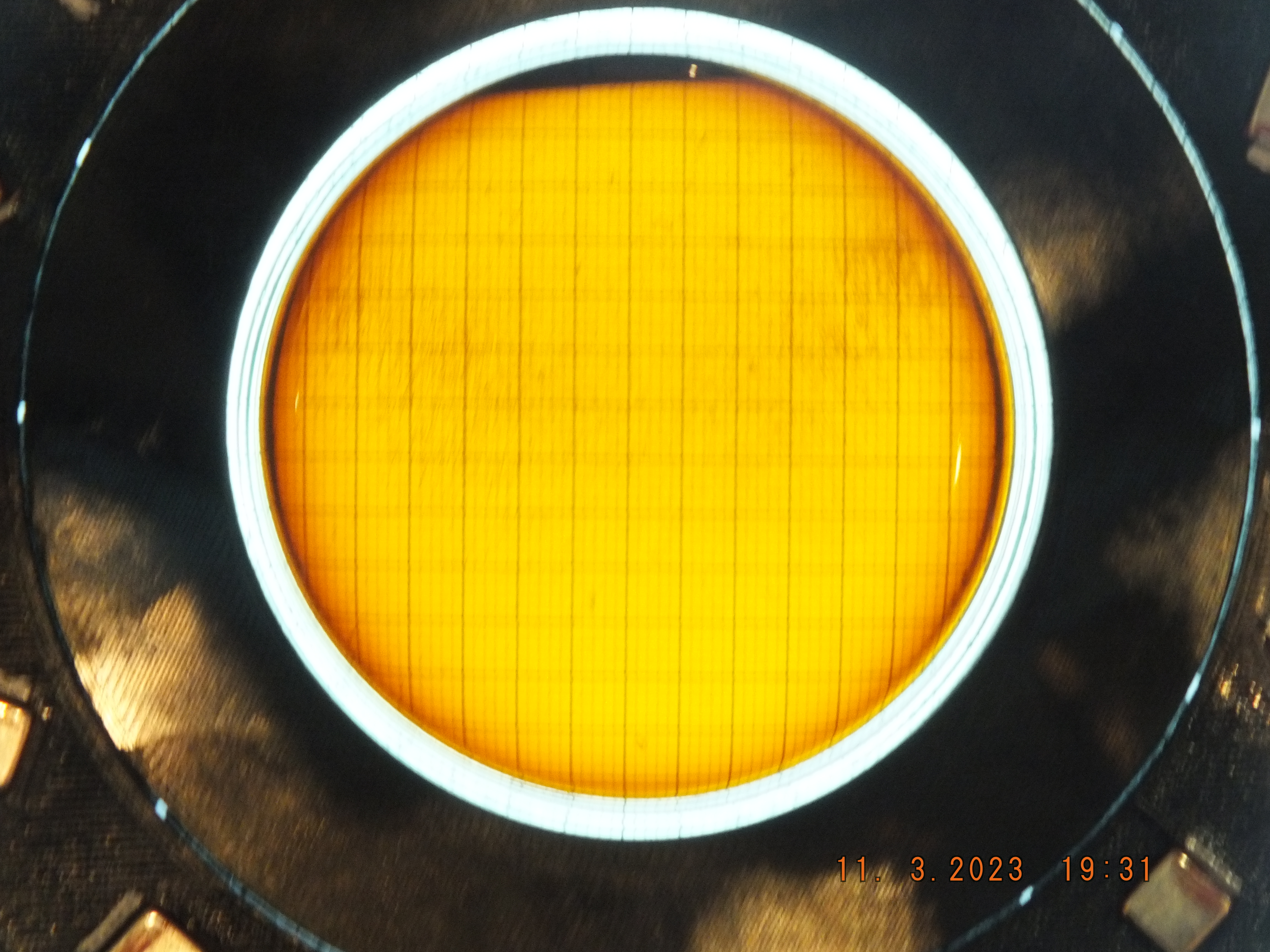

Supplement: Supplementary file 1 — Supplementary Information. [file 41598_2024_58091_MOESM1_ESM.zip › rawdata/fig7b/5_50.JPG]

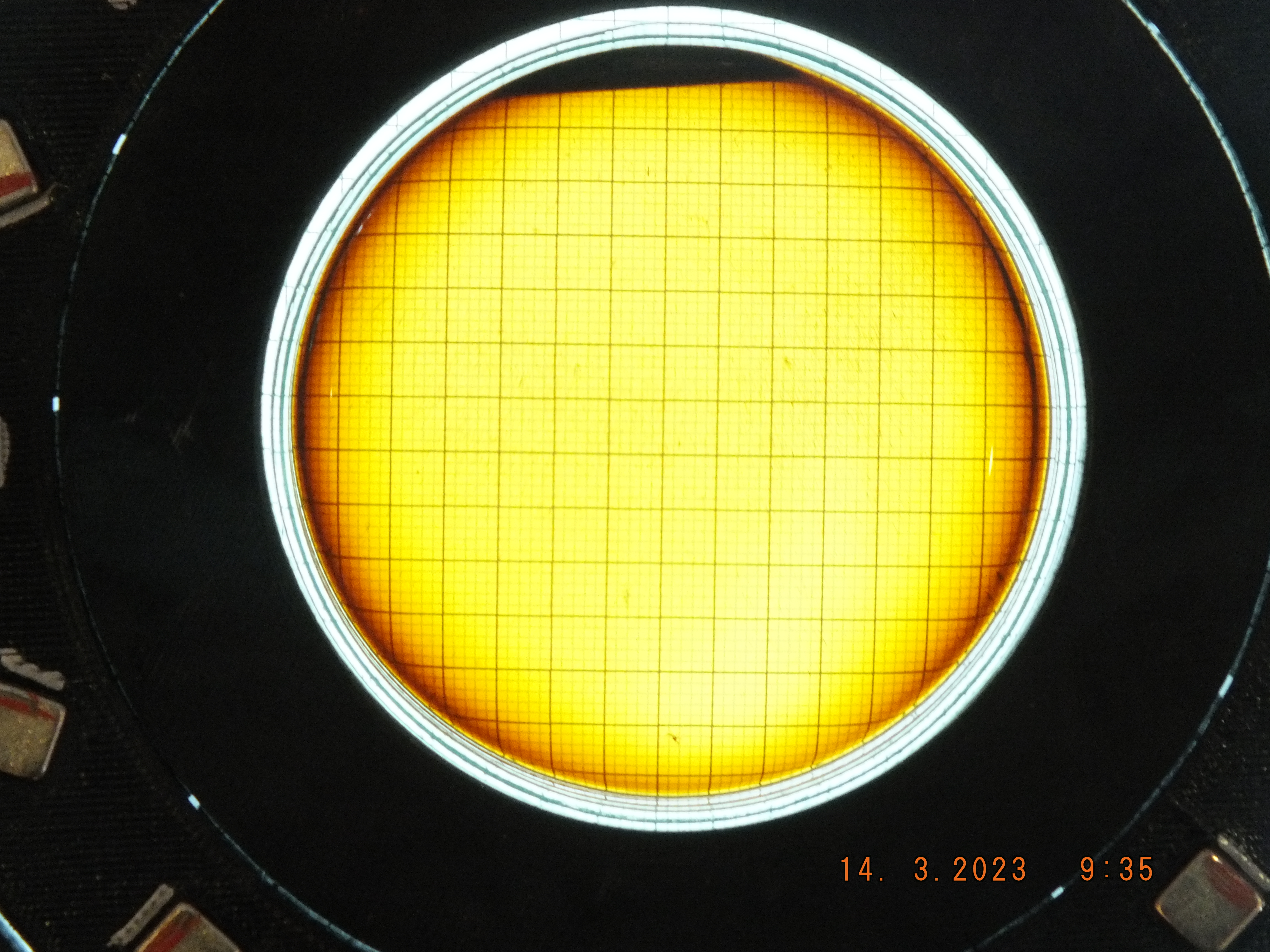

Supplement: Supplementary file 1 — Supplementary Information. [file 41598_2024_58091_MOESM1_ESM.zip › rawdata/fig7b/67_54.JPG]

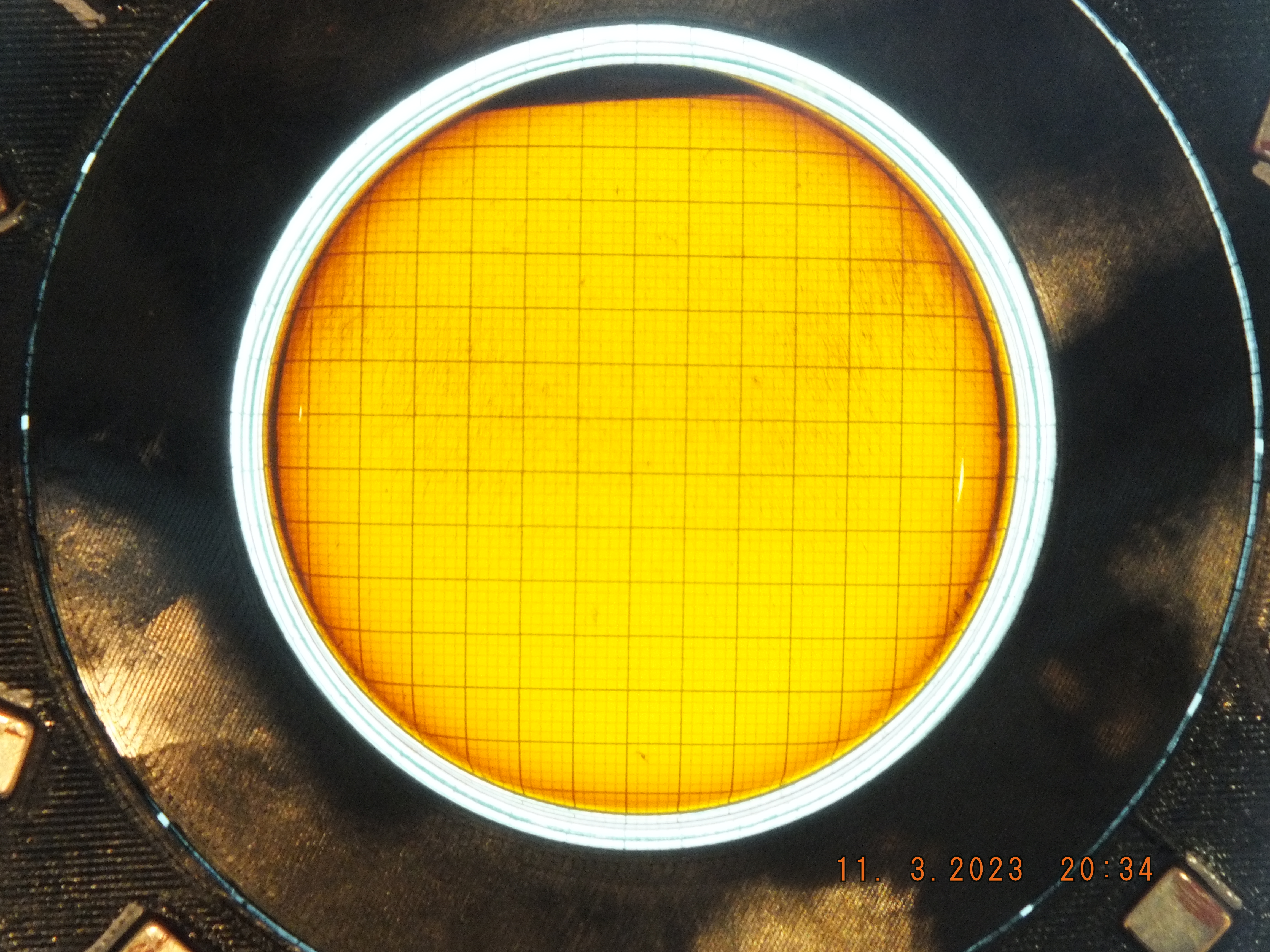

Supplement: Supplementary file 1 — Supplementary Information. [file 41598_2024_58091_MOESM1_ESM.zip › rawdata/fig7b/6_53.JPG]

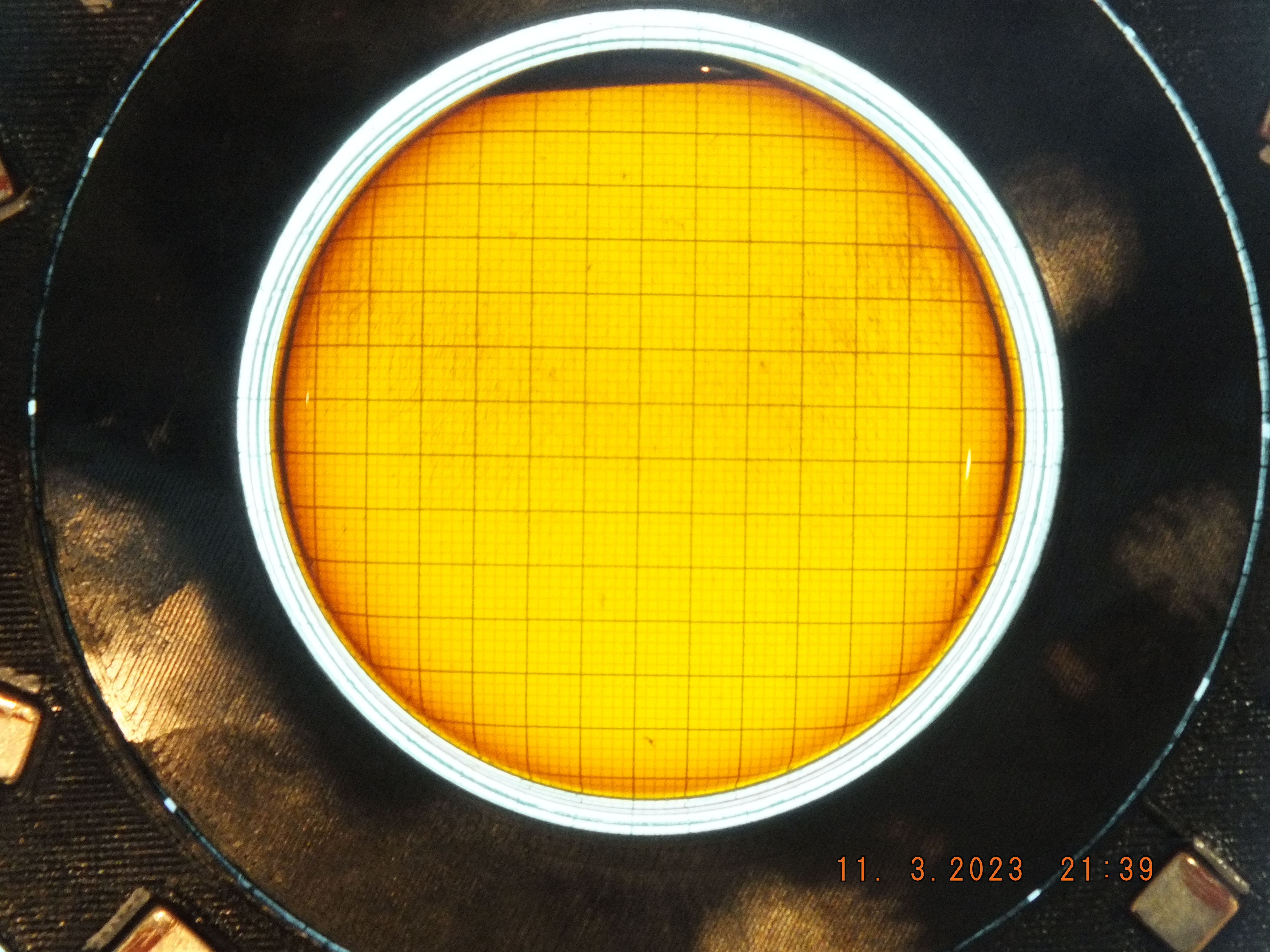

Supplement: Supplementary file 1 — Supplementary Information. [file 41598_2024_58091_MOESM1_ESM.zip › rawdata/fig7b/7_58.JPG]

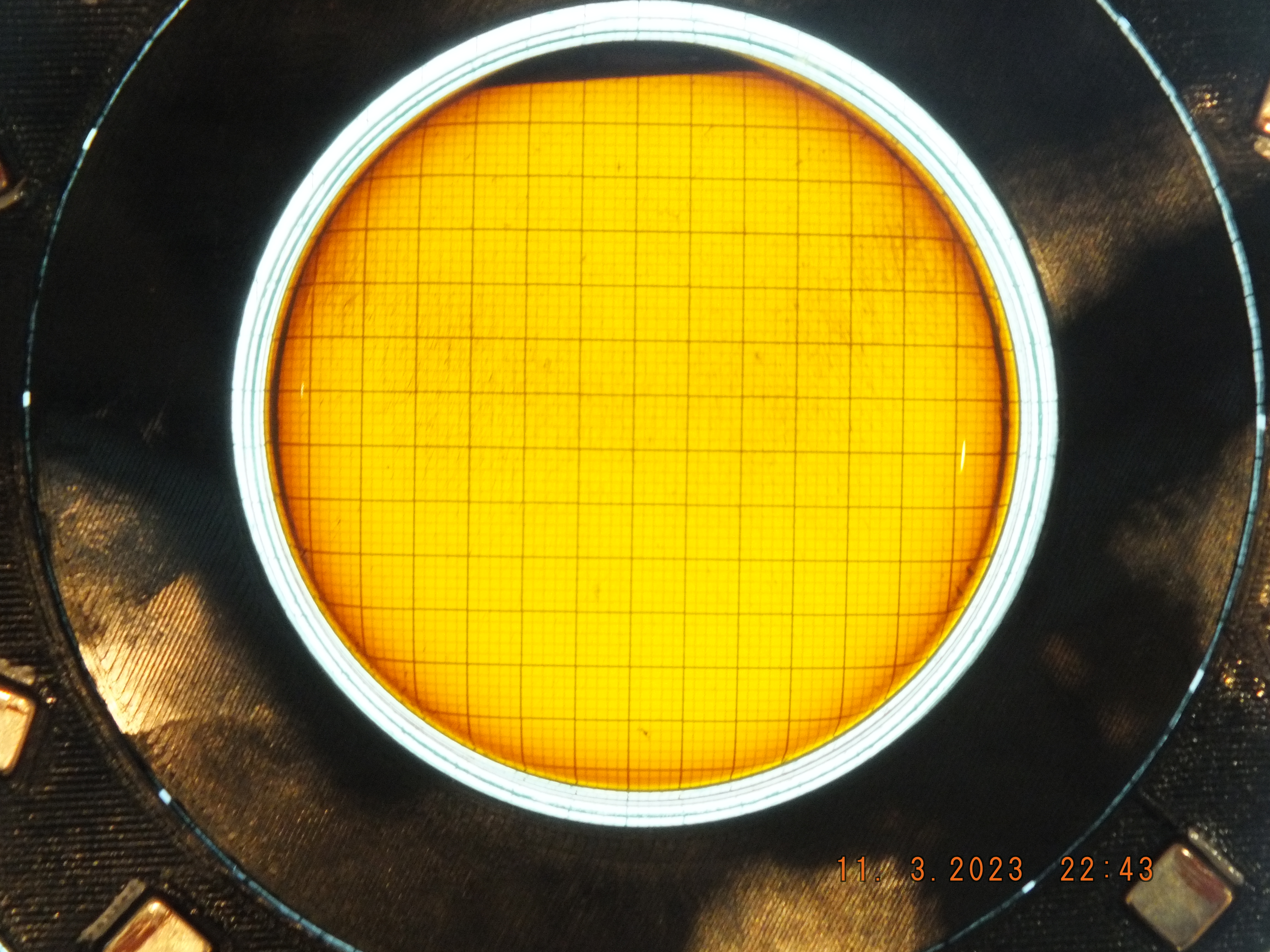

Supplement: Supplementary file 1 — Supplementary Information. [file 41598_2024_58091_MOESM1_ESM.zip › rawdata/fig7b/9_02.JPG]

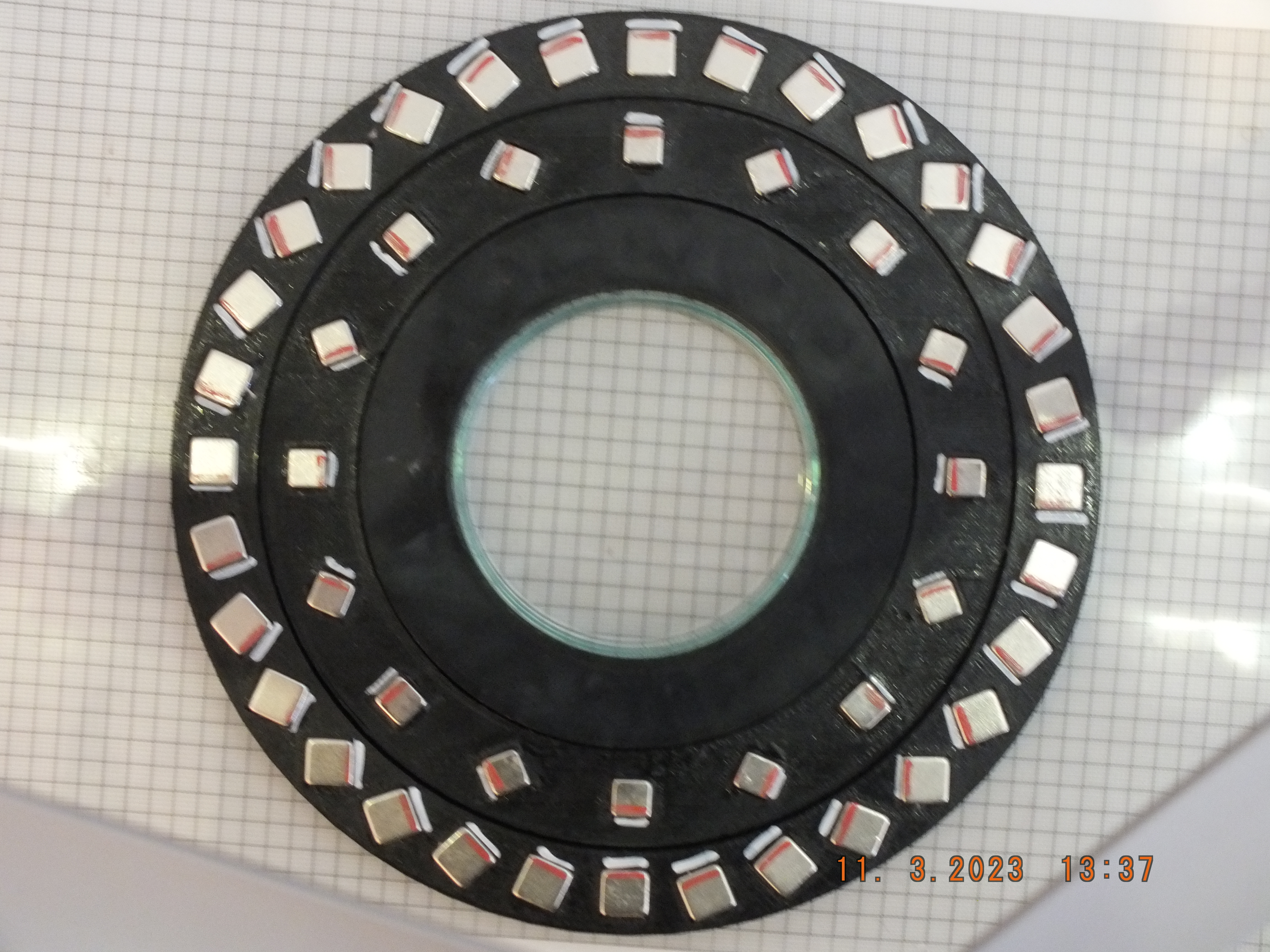

Supplement: Supplementary file 1 — Supplementary Information. [file 41598_2024_58091_MOESM1_ESM.zip › rawdata/fig7b/geometry.JPG]
